# Immune cells and their inflammatory mediators modify **β** cells and cause checkpoint inhibitor–induced diabetes

**DOI:** 10.1172/jci.insight.156330

**Published:** 2022-09-08

**Authors:** Ana Luisa Perdigoto, Songyan Deng, Katherine C. Du, Manik Kuchroo, Daniel B. Burkhardt, Alexander Tong, Gary Israel, Marie E. Robert, Stuart P. Weisberg, Nancy Kirkiles-Smith, Angeliki M. Stamatouli, Harriet M. Kluger, Zoe Quandt, Arabella Young, Mei-Ling Yang, Mark J. Mamula, Jordan S. Pober, Mark S. Anderson, Smita Krishnaswamy, Kevan C. Herold

**Affiliations:** 1 Department of Internal Medicine,; 2 Department of Immunobiology,; 3 Department of Genetics,; 4 Department of Neuroscience,; 5 Department of Computer Science,; 6 Department of Radiology and Biomedical Imaging, and; 7 Department of Pathology, Yale University, New Haven, Connecticut, USA.; 8 Department of Pathology and Cell Biology, Columbia University Irving Medical Center, New York, New York, USA.; 9 Division of Endocrinology, Diabetes, and Metabolism, Department of Internal Medicine, Virginia Commonwealth University School of Medicine, Richmond, Virginia, USA.; 10 Department of Medicine and; 11 Diabetes Center, University of California, San Francisco, San Francisco, California, USA.; 12 Huntsman Cancer Institute, University of Utah Health Sciences Center, Salt Lake City, Utah, USA.; 13 Department of Pathology, University of Utah School of Medicine, Salt Lake City, Utah, USA.

**Keywords:** Autoimmunity, Adaptive immunity, Cancer immunotherapy, Diabetes

## Abstract

Checkpoint inhibitors (CPIs) targeting programmed death 1 (PD-1)/programmed death ligand 1 (PD-L1) and cytotoxic T lymphocyte antigen 4 (CTLA-4) have revolutionized cancer treatment but can trigger autoimmune complications, including CPI-induced diabetes mellitus (CPI-DM), which occurs preferentially with PD-1 blockade. We found evidence of pancreatic inflammation in patients with CPI-DM with shrinkage of pancreases, increased pancreatic enzymes, and in a case from a patient who died with CPI-DM, peri-islet lymphocytic infiltration. In the NOD mouse model, anti–PD-L1 but not anti–CTLA-4 induced diabetes rapidly. RNA sequencing revealed that cytolytic IFN-γ^+^CD8^+^ T cells infiltrated islets with anti–PD-L1. Changes in β cells were predominantly driven by IFN-γ and TNF-α and included induction of a potentially novel β cell population with transcriptional changes suggesting dedifferentiation. IFN-γ increased checkpoint ligand expression and activated apoptosis pathways in human β cells in vitro. Treatment with anti–IFN-γ and anti–TNF-α prevented CPI-DM in anti–PD-L1–treated NOD mice. CPIs targeting the PD-1/PD-L1 pathway resulted in transcriptional changes in β cells and immune infiltrates that may lead to the development of diabetes. Inhibition of inflammatory cytokines can prevent CPI-DM, suggesting a strategy for clinical application to prevent this complication.

## Introduction

Checkpoint inhibitors (CPIs), which block immune-inhibitory ligand-receptor interactions, have revolutionized cancer treatment due to their efficacy and ability to improve survival in a growing number of cancer types (1, 2). Cytotoxic T lymphocyte antigen 4 (CTLA-4) and programmed death 1 (PD-1) are checkpoint molecules essential for maintaining immunologic tolerance by impeding T cell activation (3). While monoclonal antibodies (mAbs) against CTLA-4, PD-1, and its ligand, programmed death ligand 1 (PD-L1), increase tumor cell destruction, they can also lead to loss of self-tolerance. This can result in immune-related adverse events (irAEs) affecting various organ systems including pancreatic β cells and autoimmune diabetes (4–10).

CPI-induced diabetes mellitus (CPI-DM) is a relatively uncommon complication of CPI therapy with an estimated overall prevalence of 0.2% to 1.9% of CPI-treated individuals but can present acutely and may be life-threatening (7, 8, 10–14). CPI-DM occurs almost exclusively with anti–PD-1 and anti–PD-L1 treatment alone or in combination with anti–CTLA-4 treatment (10, 12, 15, 16). This form of insulin-dependent diabetes is clinically similar to type 1 diabetes (T1D) with acute metabolic decompensation, severe hyperglycemia, diabetic ketoacidosis, and low C-peptide, suggesting significant β cell loss, but there are unique features, such as a lower incidence of autoantibodies (~40%), an increased frequency of HLA-DR4 (>70%), and more rapid loss of β cells (i.e., measurable C-peptide levels) (8, 10, 11, 17–24). The underlying mechanisms of this emerging form of diabetes, and selectivity for PD-1/PD-L1 blockade, remain largely unknown. Herein, we investigated the inflammatory and islet cell changes that occur in CPI-DM to understand the mechanisms and differential effects of CPIs on the development of diabetes.

Through its negative costimulatory actions on effector and regulatory pathways, PD-1/PD-L1 has been implicated in immune tolerance and autoimmunity in NOD mice (25–31). PD-1 or PD-L1 deficiency leads to significantly accelerated diabetes, and overexpression of PD-L1 can prevent diabetes in NOD mice (32–38). A genome-wide expression profiling study revealed upregulation of the PD-L1 gene (*CD274*) in peripheral blood samples from new-onset T1D (39). *CD274* gene polymorphisms in T1D and low serum levels of PD-L1 in individuals with T1D have also been described (40).

Although insulin deficiency is a key feature of CPI-DM, the details of the inflammation present in the pancreas remain unclear. We and others have previously described that patients with CPI-DM exhibit increased levels of lipase and/or amylase prior to or at the time of diabetes diagnosis, indicating pancreatic inflammation (8, 41–45). While the inflammatory mediators that are seen with pancreatitis, IFN-γ, IL-1β, TNF, and IL-6 (46, 47), may provoke tissue pathology, they can also induce the expression of immune-inhibitory ligands as an opposing mechanism that may protect β cells from killing. PD-L1 expression increases on β cells in NOD mice with infiltration of immune cells and progression of diabetes (48, 49). Recently PD-L1 was found on human pancreatic β cells in response to IFN-α and IFN-γ and in islets of patients with T1D (48, 50). The *CD274* promoter has known IFN regulatory factor 1 (IRF1) binding sites, and the JAK/STAT/IRF1 signaling pathway is a known regulator of PD-L1 expression and appears to be involved in the regulation of PD-L1 on β cells (50, 51). However, these inflammatory mediators may also play a pathologic role directly and indirectly, suggesting a more complicated relationship between PD-L1 expression and responses to inflammation on human β cells beyond inhibition of effector T cells.

To understand the pancreatic tissue and immune cell interactions that may lead to β cell killing in patients treated with CPIs, we analyzed clinical, cellular, and molecular changes that occur in β cells in the setting of CPI in humans and in NOD mice with anti–PD-L1–induced diabetes. We found evidence of pancreatic inflammation in patients with CPI-DM and expression of checkpoint molecules in pancreatic tissue and on β cells from a patient who died with CPI-DM. RNA sequencing of islets from anti–PD-L1– versus anti–CTLA-4–treated NOD mice identified characteristics of immune cells and β cells unique to anti–PD-L1 treatment and enhanced by inflammatory pathways. Our studies of human islets show how death pathways may be activated by IFN-γ. Finally, we demonstrate that blockade of inflammatory pathways can prevent CPI-DM in NOD mice, which has potential clinical and therapeutic implications.

## Results

### Exocrine pancreas inflammation in patients with or without CPI-induced diabetes.

**Figure 1:**
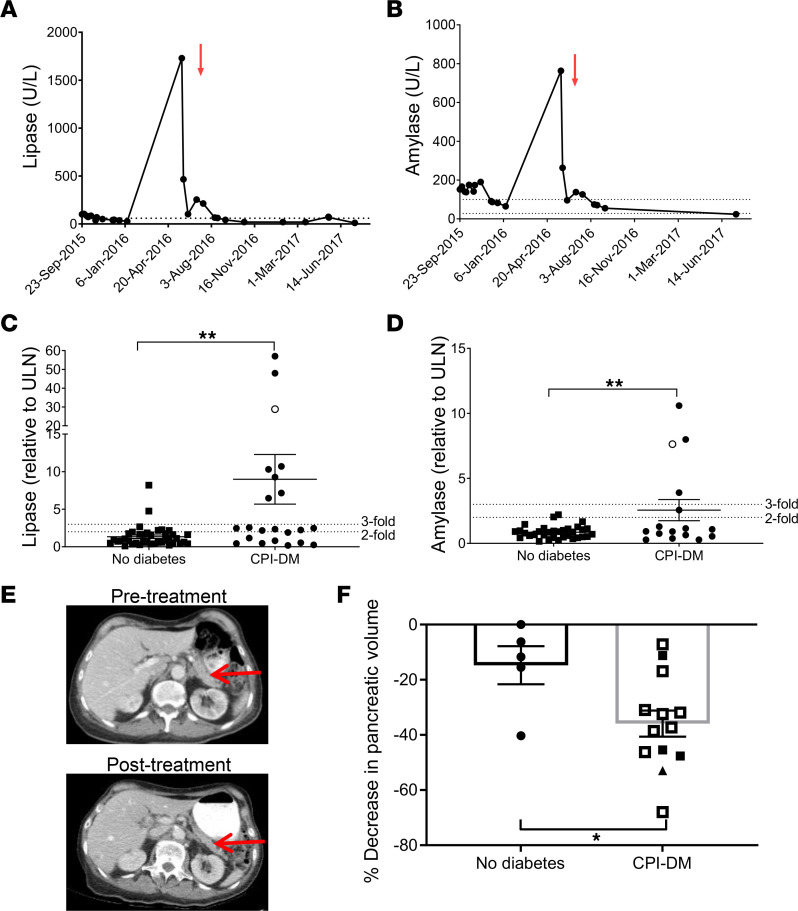
Exocrine pancreas inflammation in patients with or without CPI-induced diabetes. (**A**) Lipase and (**B**) amylase levels in a patient who developed CPI-DM (diagnosed at the time indicated by the red arrow). (**C**) Lipase and (**D**) amylase levels were increased in patients who developed CPI-DM compared with CPI-treated patients who did not develop diabetes following treatment. Mean fold induction (SEM) above upper limit of normal (ULN) for lipase 1.34 (0.23) (*n* = 39) versus 8.99 (3.30) (*n* = 22) for control and CPI-DM, respectively, and amylase 0.86 (0.08) (*n* = 33) versus 2.56 (0.81) (*n* = 16). Student’s 2-tailed *t* test, **P ≤ 0.01. The patient in **A** and **B** is indicated by the unfilled circle. (**E**) CT scans of a patient with CPI-DM before and after CPI treatment. The posttreatment scan was obtained 4 days prior to diabetes onset, which occurred 25 weeks from CPI initiation. The red arrow identifies the pancreas. (**F**) Pancreatic volume, calculated from abdominal CT scans before and after CPI therapy, showed a significant reduction after CPI treatment in patients with CPI-DM (*n* = 13) compared with patients without CPI-DM (*n* = 5) (mean [SEM]) (35.9 [4.75] versus 14.8 [6.90] percentage reduction compared with pretreatment volumes respectively, Student’s 2-tailed *t* test, *P* = 0.029). Solid circles or squares = lipase < 2-fold ULN. Empty circles or squares = lipase > 2-fold ULN. Triangle indicates patient in **E**.

We analyzed the clinical features and chemistries from 61 patients with cancers who were treated with CPIs. Of these, 22 had developed CPI-DM after a median of 22 weeks (range 2–81 weeks) from initiation of CPI treatment. Lipase levels were measured a median of 15 weeks (range 2 weeks to 83 weeks) from CPI start in those who developed diabetes and 16 weeks (range 1 week to 173 weeks) in those who did not develop diabetes (NS by unpaired Student’s *t* test) (Supplemental Table 1; supplemental material available online with this article; https://doi.org/10.1172/jci.insight.156330DS1). In those who developed diabetes, lipase levels were measured from 20 weeks before to 18 weeks after the time of diabetes diagnosis. The lipase levels were increased above upper limit of normal 1.34 ± 0.23 (*n* = 39) versus 8.99 ± 3.30 (*n* = 22) fold for control and CPI-DM, respectively (*P* < 0.01), and amylase: 0.86 ± 0.08 (*n* = 33) versus 2.56 ± 0.81 (*n* = 16) (*P* < 0.01) fold (Figure 1, A–D). There were more patients with at least a 3-fold increase of lipase among those who developed diabetes than those who did not (36.4% [8/22] in diabetics versus 5.1% [2/39] in controls, Fisher’s exact test *P* = 0.003). Only 1 patient in the diabetes group and 1 patient in the control group had clinical symptoms of pancreatitis.

To determine the significance of these biochemical findings, we compared the pancreatic volume, calculated from abdominal CT scans, before and after CPI therapy. There was a significant reduction in pancreatic volume after CPI treatment in patients with CPI-DM compared with CPI-treated patients who did not develop diabetes (35.9% ± 4.75% versus 14.8% ± 6.90% reduction compared with pretreatment volumes, respectively) (unpaired Student’s *t* test, *P* = 0.029) (Figure 1, E and F).

**Figure 2:**
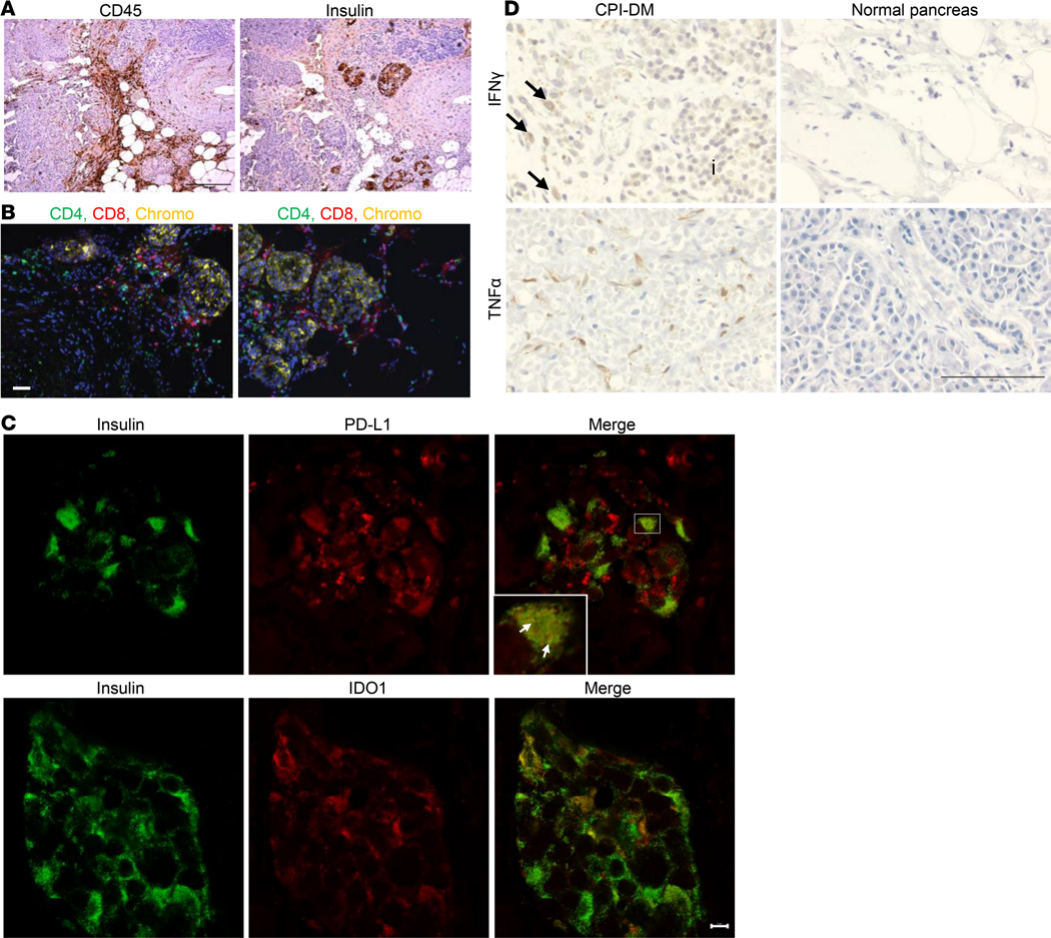
Evidence of inflammation in pancreatic tissue from a patient with CPI-DM. (**A**) Immunohistochemistry staining of pancreatic tissue from a patient with CPI-DM shows evidence of CD45^+^ lymphocytic infiltrates in areas surrounding islets. The tissue was obtained 25 days from diabetes diagnosis and 42 days from CPI start. Scale bar: 100 μm. (**B**) Immunocytochemistry staining of pancreatic tissue from a patient with CPI-DM shows CD4^+^ T cell and CD8^+^ T cells infiltrating in a peri-islet distribution. Scale bar: 50 μm. (**C**) Confocal images demonstrating PD-L1 and IDO1 expression in β cells in the patient with CPI-DM. Scale bar: 5 μm. Inset shows higher magnification image (3.3×) of islet with colocalization of PD-L1 and insulin expression on β cells (white arrows). (**D**) Immunohistochemistry staining of the pancreatic tissue from the patient with CPI-DM shows IFN-γ (IFN-γ^+^ mononuclear cells indicated with black arrows) in mononuclear cells within and surrounding islets (indicated with “i”) and TNF-α staining in stromal cells. IFN-γ and TNF-α were not detected in pancreases from 2 normal individuals. Scale bar: 100 μm.

Our understanding of the pancreatic tissue changes that occur in patients with CPI-DM remains sparse due to limited access to pancreatic tissue from these patients. We obtained pancreatic tissue from the autopsy of a patient with CPI-DM after the patient died from myocarditis. This 78-year-old woman without prior history of diabetes developed CPI-DM approximately 17 days after receiving 1 dose of durvalumab (anti–PD-L1) and tremelimumab (anti–CTLA-4) for myelodysplastic syndrome. Her amylase levels were 2.23-fold above the upper limit of normal, but the lipase was normal at the time of diabetes diagnosis. We found inflammatory cells (CD45^+^) in exocrine tissue in areas surrounding islets (Figure 2A) and CD4^+^ and CD8^+^ T cells in a peri-islet distribution (Figure 2B). Both PD-L1 and indoleamine 2,3 dioxygenase-1 (IDO1) were expressed in β cells (Figure 2C). To explore whether the expression of these immune-inhibitory ligands is a general feature of pancreatic inflammation or whether it is specific for CPI-DM, we stained pancreatic tissue from patients with autoimmune and chronic pancreatitis (Supplemental Figure 1A) and also found PD-L1 (Supplemental Figure 1B) and IDO1 (Supplemental Figure 1C) expression in β cells in these inflammatory conditions.

The toxic effects of IFN-γ and TNF are thought to play a role in the pathogenesis of pancreatitis (52). To confirm these findings in vivo, we performed immunohistochemical staining to identify cytokines in the pancreas from the patient who died with CPI-DM. We found IFN-γ and TNF-α expression in cells within peri-islet inflammatory infiltrates and the stroma from the patient (Figure 2D).

### Differential effects of checkpoint blockade on diabetes and immune cells in mice treated with checkpoint inhibitors.

**Figure 3:**
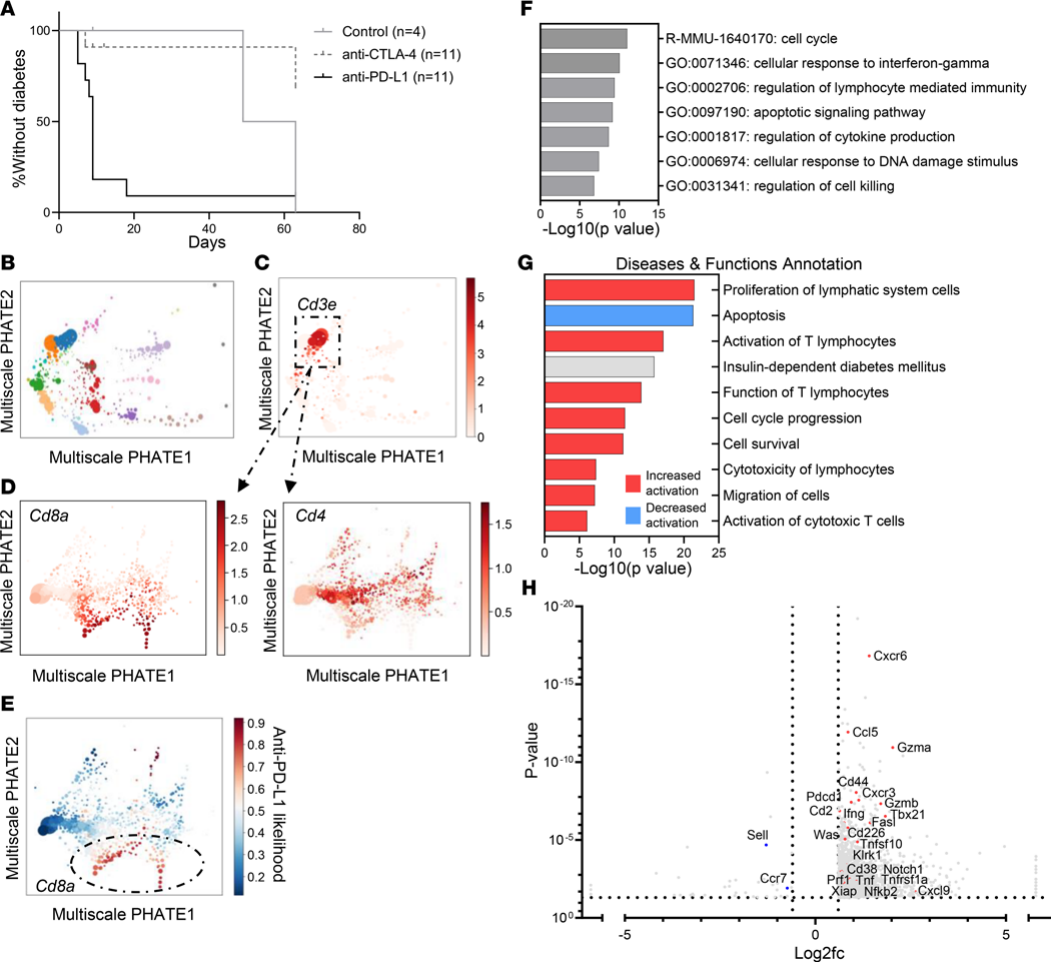
Single-cell RNA-Seq of CD8^+^ T cells from anti–PD-L1 versus anti–CTLA-4 mAb–treated NOD mice. (**A**) NOD mice at 7 weeks old treated with anti–PD-L1 (*n* = 11) rapidly develop diabetes whereas anti–CTLA-4–treated mice (*n* = 11) and age-matched control NOD mice (*n* = 4) do not. (*P* = 0.002, log-rank test [Mantel-Cox test].) (**B**) Immune and islet cell populations were identified by Multiscale PHATE analysis. (**C**) Multiscale PHATE analysis highlighting T cells (CD3E^+^) from all 14,892 cells treated with anti–CTLA-4 and anti–PD-L1. (**D**) Among the CD3E^+^ T cells, CD8^+^ T cells and CD4^+^ T cells are present in the islets from anti–CTLA-4– and anti–PD-L1–treated mice. (**E**) MELD analysis of T cells indicating that differences in CD8^+^ T cells were associated with anti–PD-L1 versus anti–CTLA-4 mAb treatment. (**F**) Metascape analysis of differentially expressed genes (1,039 genes with *P* < 0.05, *q* < 0.05, log2fc ≤ –0.6 and ≥ 0.6) between CD8^+^ T cells from anti–PD-L1 or anti–CTLA-4 mAb–treated mice revealed differences in pathways regulating cell cycle, responses to IFN-γ, apoptosis/cell killing, and cytokine production. (**G**) Diseases and functions predicted to be impacted with CPI treatment in CD8^+^ T cells by IPA. Predicted activation state is for anti–PD-L1 CD8^+^ T cells compared with anti–CTLA-4 CD8^+^ T cells. Activation *z* score cutoffs ≤ –2 and ≥ 2. (**H**) Select differentially expressed genes in CD8^+^ T cells consistent with a cytotoxic phenotype with anti–PD-L1 treatment. *Cxcr3*, *Ifng*, *Pdcd1*, *Gzmb/a*, and *Fasl* were among those genes elevated in CD8^+^ T cells in anti–PD-L1–treated mice. Volcano plot includes 2,007 genes based on *P* < 0.05, *q* < 0.05, log2fc ≤ –0.6 and ≥ 0.6 with genes of interest highlighted in red or blue. log2fc, log_2_ fold change.

To identify the relationships between immune and β cell changes in the pancreatic islets, we utilized the prediabetic NOD mouse model. Diabetes was precipitated by anti–PD-L1 but not anti–CTLA-4 mAb treatment in 7-week-old NOD mice, consistent with our clinical experience of the selectivity of CPI-DM following PD-1 pathway blockade and previous studies on PD-1 blockade in the model (Figure 3A) (37). Although diabetes only occurred following anti–PD-L1 treatment, both anti–PD-L1–treated NOD mice and anti–CTLA-4–treated NOD mice developed immune infiltrates (Supplemental Figure 2, A–C), indicating that differences in the immune and/or β cells account for the susceptibility to diabetes.

We analyzed endocrine (i.e., CD45^–^) and immune cells (i.e., CD45^+^) from islets from anti–PD-L1–treated NOD mice and anti–CTLA-4–treated NOD mice that were isolated after 2 doses of antibody, by bulk RNA sequencing (RNA-Seq). For comparison, we also analyzed islet and immune cells from untreated 11-week-old NOD mice to compare with cells from spontaneous autoimmune diabetes.

The expression of 285 genes differed significantly between CD45^+^ cells from anti–CTLA-4– and anti–PD-L1–treated NOD mice (Supplemental Figure 3, A and B). Gene set enrichment showed that both IFN-β– and IFN-γ–responsive genes were higher in anti–PD-L1– versus anti–CTLA-4- treated mice (Supplemental Figure 3C). *Ifng* expression was 2-fold higher in anti–PD-L1–treated CD45^+^ cells compared with anti–CTLA-4–treated mice (*P* = 8.4 × 10^–3^, 2.07-fold change, FDR step-up 0.18) (Supplemental Table 2). Genes involved in cytolytic cellular responses were significantly higher with anti–PD-L1 treatment: *Gzmb*, *Gzma*, *Fasl*, as well as *Cd8a* (Supplemental Figure 3B and Supplemental Table 2). Chemokines (*Cxcl9*) and immune ligands (*Pdcd1*) were increased in the immune cells from the anti–PD-L1 versus anti–CTLA-4 mAb–treated mice, consistent with activation of T cells in the islets of anti–PD-L1–treated mice. Comparison of anti–PD-L1–treated and untreated NOD mice revealed overlap in expression of 116 genes that were also different between anti–PD-L1 and anti–CTLA-4, such as those involved in cytokine-cytokine receptor interactions, including *Cxcl9*, *Cxcl10*, *Ifng*, and *Cd40*, as well as *Pdcd1* and cytotoxic genes, such as *Gzmb* and *Gzma* (Supplemental Figure 3, B and E).

In islet cells, there were 31 differentially expressed genes from anti–PD-L1– versus anti–CTLA-4–treated mice (Supplemental Figure 4, A–C, and Supplemental Table 3). These were in IFN-γ response pathways, such as *Cd274* and *Irf1*, as well as the IFN-γ–induced chemokine *Cxcl10*. Gene expression was confirmed by quantitative PCR (qPCR) for immune and islet cells (Supplemental Figure 3D and Supplemental Figure 4D, respectively).

**Figure 4:**
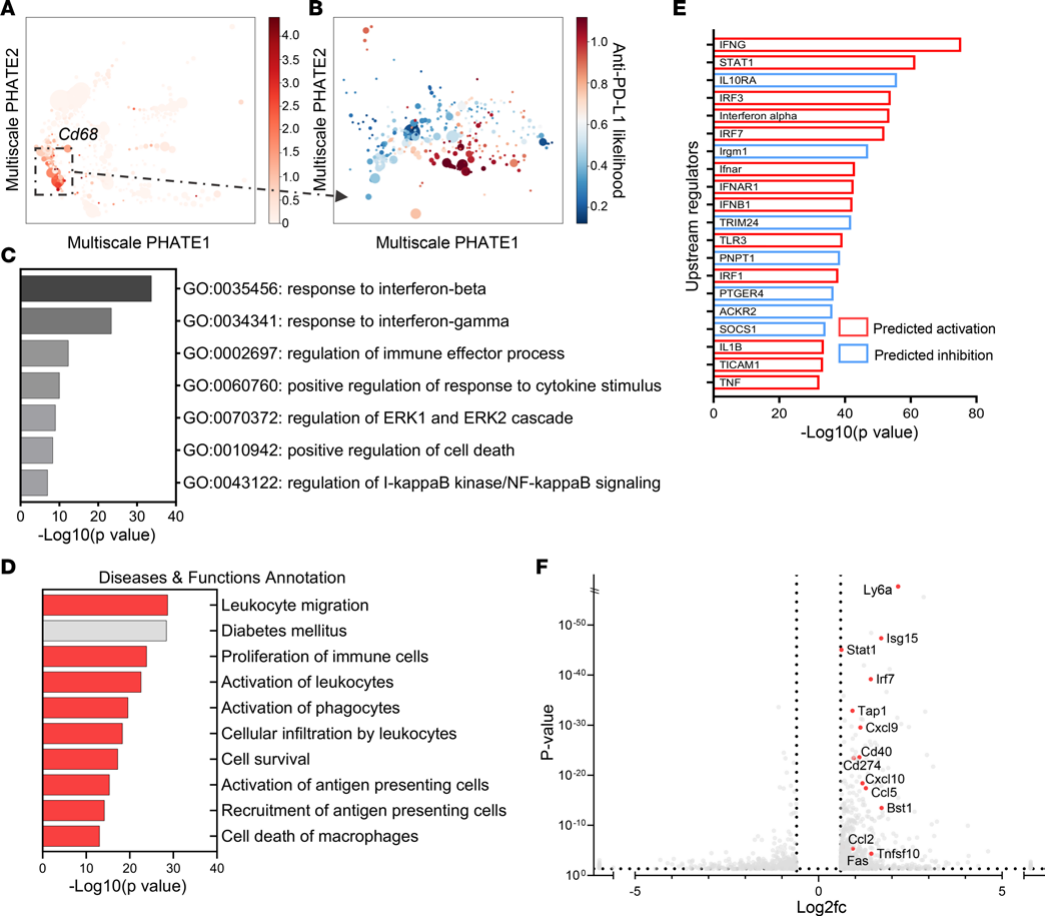
Differentially expressed pathways and genes in islet-infiltrating macrophages with anti–PD-L1 versus anti–CTLA-4 mAb treatment. (**A**) Macrophages (CD68^+^) are highlighted on Multiscale PHATE visualization of all cells treated with anti–CTLA-4 and anti–PD-L1. (**B**) MELD analysis of macrophage subpopulations showed differences between anti–PD-L1 versus anti–CTLA-4 treatment conditions. (**C**) Metascape analysis of differentially expressed genes by scRNA-Seq between anti–PD-L1–treated and anti–CTLA-4–treated islet-infiltrating macrophages. (711 genes based on *P* < 0.05, *q* < 0.05, log2fc ≤ –0.6 and ≥ 0.6). (**D**) Diseases and functions represented by differentially expressed genes between anti–PD-L1– and anti–CTLA-4–treated macrophages by Ingenuity Pathway Analysis (IPA; QIAGEN). Predicted activation state is for anti–PD-L1 macrophages compared with anti–CTLA-4 macrophages. Activation *z* score cutoffs ≤ –2 and ≥ 2. Red = predicted to be activated, and gray = significant change without defined direction. (**E**) Top 20 predicted upstream regulators in macrophages by IPA. Red = upregulated in anti–PD-L1 macrophages, and blue = downregulated in anti–PD-L1–treated macrophages. (**F**) Volcano plot of differentially expressed genes in anti–PD-L1 versus anti–CTLA-4 treatment macrophages. Highlighted genes include IFN-γ–responsive genes. There were 1,329 differentially expressed genes based on *P* < 0.05, *q* < 0.05 (not shown), log2fc ≤ –0.6 and ≥ 0.6.

To identify which cells accounted for these differences, we performed single-cell RNA-Seq (scRNA-Seq) of islets from anti–PD-L1– and anti–CTLA-4–treated NOD mice. We visualized the cells with Multiscale Potential of Heat-diffusion for Affinity-based Transition (PHATE), which preserves manifold structure, to identify cell populations and transitions, and used the MELD algorithm to highlight islet cells that were enriched/depleted when the 2 treatment conditions were compared (Figure 3, B–E, and Figure 4A). The differences in CD8^+^ and CD68^+^ cells were greatest with this analysis (Figure 3E and Figure 4, A and B). In CD8^+^ T cells, pathway analysis of differentially expressed genes between anti–CTLA-4 and anti–PD-L1 islet-infiltrating CD8^+^ T cells included those regulating cellular responses to IFN-γ, leukocyte proliferation and activation, cytokine production, apoptotic signaling pathways, and lymphocyte cytotoxicity and migration (Figure 3, F and G). IL-2 was predicted to be an upstream regulator in CD8^+^ T cells from anti–PD-L1 versus anti–CTLA-4 treatment, and *Il2ra* gene expression was higher (*P* = 0.0016, *q* = 0.02, log2fc = 1.2). Anti–PD-L1 treatment was also associated with a decrease in apoptosis and increased cell survival pathways in CD8^+^ T cells compared with anti–CTLA-4 treatment (Figure 3G). Consistent with our bulk RNA-Seq data, genes of cytotoxic CD8^+^ T cells were increased in anti–PD-L1–treated mice (*Gzma*, *Gzmb*, *Fasl*, *Prf1*, *Ifng*, *Tnf*, and *Cxcr3*) (Figure 3H).

In the macrophages in insulitis, identified by *Cd68* gene expression, we found differentially expressed genes in anti–PD-L1 versus anti–CTLA-4 treatment–enriched subpopulations (Figure 4, A and B). IFN-γ response pathway genes, regulation of immune effector processes, and leukocyte migration and recruitment were enriched in the anti–PD-L1 treatment group (Figure 4, C–E). In addition, expression of *Cd274*, *Stat1*, *Cxcl9*, and *Cxcl10* (Figure 4F) was increased in macrophages with anti–PD-L1 treatment. These findings are consistent with recent findings by Hu et al. demonstrating a role for activated macrophages responding to IFN-γ in diabetes induced by blockade of PD-1 (53). Immunohistochemistry staining of pancreatic tissue from anti–PD-L1 and anti–CTLA-4–treated mice confirmed in vivo expression of inflammatory mediators in the anti–PD-L1–treated NOD mice (Supplemental Figure 5).

### Atypical features of β cells from anti–PD-L1–treated NOD mice.

**Figure 5:**
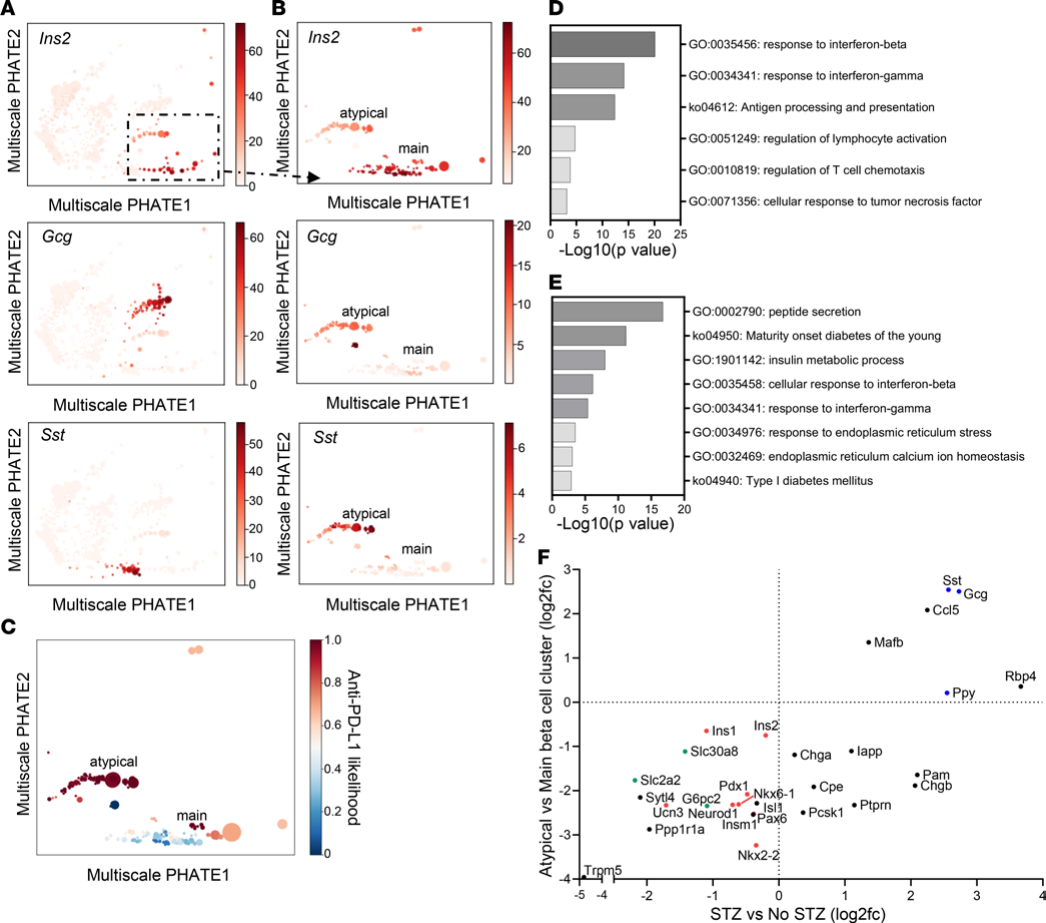
Changes in β cells are identified with anti–PD-L1 treatment. (**A**) Visualization of main islet cell populations by Multiscale PHATE: β cells (high *Ins2*), α cells (high *Gcg*), and δ cells (high *Sst*) are highlighted. There were 233 β cells from anti–CTLA-4 treatment and 150 β cells from anti–PD-L1 treatment in the main β cell cluster and 393 β cells from anti–PD-L1 treatment in the atypical β cell cluster. (**B**) Analysis of the high *Ins2*^+^ cells reveals an atypical β cell population identified with anti–PD-L1 treatment only that expresses lower *Ins2* and higher *Gcg* and *Sst* than the main β cell population. (**C**) MELD analysis shows the association of the atypical β cell subpopulation with anti–PD-L1 treatment. (**D**) Differential expression of genes in pathways including IFN responses, antigen processing and presentation, regulation of lymphocyte function, and TNF-α responses, upregulated in β cells with anti–PD-L1 treatment compared with anti–CTLA-4 treatment, within the main β cell cluster. Pathway analysis of 134 genes based on *P* < 0.05, *q* < 0.05, log2fc ≤ –0.6 and ≥ 0.6. (**E**) Differential expression of genes in pathways including maturity-onset diabetes of the young (MODY), T1D, peptide/insulin processing, and ER function were different between β cells in the atypical β cell cluster compared with the main β cell cluster in anti–PD-L1–treated islets. (**F**) Comparison of differentially expressed genes between the atypical β cell cluster versus the main β cell cluster and streptozotocin (STZ) versus no STZ from Sachs et al. by log2fc (54). Highlighted genes include those involved in β cell identity/maturity (red), insulin secretion (green), and other islet cell markers (blue).

We identified and compared islet cells from our treatment conditions with the 2 mAbs using Multiscale PHATE and MELD. We identified 2 β cell clusters in mice that were treated with anti–PD-L1 mAb whereas only a single cluster was found with anti–CTLA-4 mAb (Figure 5, A–C). Both populations were β cells by virtue of high levels of expression of *Ins1/2*. In β cells we found differential expression of genes in pathways of IFN responses, antigen processing and presentation, regulation of lymphocyte activation, regulation of lymphocyte chemotaxis, and cellular response to TNF when the cells were compared from anti–PD-L1 and anti–CTLA-4 mAb–treated mice (Figure 5D). Upstream regulators predicted to be activated with anti–PD-L1 treatment include STAT1, IFN-γ, and TNF-α (Supplemental Figure 6). Consistent with bulk RNA-Seq data, IFN-γ–responsive genes were upregulated on β cells with anti–PD-L1 treatment, including *Cxcl10*, *Cd274*, *Stat1*, and *Irf1* (Supplemental Table 4).

The atypical β cell cluster in the anti–PD-L1–treated mice expressed genes involved in pathways of maturity-onset diabetes of the young (MODY) as well as T1D, insulin processing, and ER function (Figure 5E). This cluster of β cells expressed lower *Ins1/2*, *Chga*, *Mafa*, and *Nkx6*.*1* and higher levels of *Gcg*, *Sst*, and *Cxcl9* (Table 1).

We and others have described β cell clusters during the development of diabetes that have features of dedifferentiation similar to those we had found following anti–PD-L1 mAb treatment (49). Therefore, we compared our differentially expressed genes in the atypical β cell cluster to the immature/dedifferentiated β cells observed in the streptozotocin treated diabetic mouse model in Sachs et al. (54). There was a significant correlation in the top 75 differentially expressed genes for both data sets (Spearman *r* = 0.69, *P* = 0.0186). Both data sets identified differences in genes of the pathways of MODY, peptide secretion/insulin metabolism, and ER stress. Like our β cells, they identified lower expression of β cell identity/maturity, such as *Ins1*, *Ins2*, *Ucn3*, *Pdx1*, *Nkx6.1*, *Nkx2.2*, *Pax6*, and *Neurod1*; lower expression of genes involved in insulin secretion, such as *Slc2a2*, *Slc30a8*, and *G6pc2*; and higher expression of other islet cell markers, such as *Gcg* and *Sst* (Figure 5F). These features are consistent with dedifferentiation that may occur in response to immunologic stressors.

### IFN-γ induces changes in human β cells, including expression of cell death pathways.

**Figure 6:**
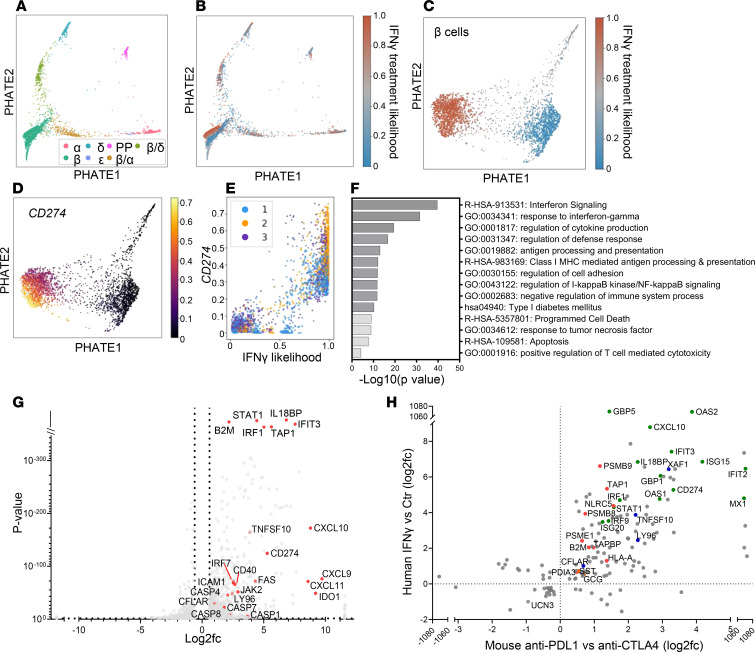
Transcriptional changes in human islets in response to IFN-γ. (**A**) PHATE analysis of scRNA-Seq of control and IFN-γ–treated human β cells. Human islets from 3 donors treated for 24 hours in the presence or absence of 25 ng/mL IFN-γ were sorted using TMRE and FluoZin-3 for β cell enrichment and subsequently analyzed by 10x Genomics. PHATE analysis shows distribution of islet cells from the 3 donors. (**B** and **C**) MELD revealed distinct populations of β cells in the presence and absence of IFN-γ. Separation of β cells compared with other islet cell populations in **B** indicates a greater impact of IFN-γ treatment on those cells. (**D**) PHATE analysis shows higher expression of *CD274* in IFN-γ–treated β cells and (**E**) MELD identified a transition in β cells that is accompanied by a change from low to high *CD274* (*R*^2^ = 0.87). (**F**) Pathway analysis of differentially expressed genes between control and IFN-γ–treated β cells reveals differences in pathways involved in IFN signaling (IFN-γ), cytokine production, antigen processing and presentation, apoptosis, and responses to TNF-α. (**G**) Volcano plot of differentially expressed genes between control and IFN-γ–treated β cells (2,134 genes based on *P* < 0.05, *q* < 0.05, log2fc ≤ –0.6 and ≥ 0.6), highlighting genes involved in IFN signaling, chemokines, and regulators of apoptosis. (**H**) Correlation of the overlapping 144 genes (among the top 1,000 differentially expressed genes in β cells) in the anti–PD-L1–treated mouse scRNA-Seq data set and the human islet IFN-γ treatment data set shown as log2fc. Highlighted genes include those involved in IFN-γ response (green), antigen processing/presentation (red), and cell death pathways (blue).

The inflammatory mediators that we identified in CPI-DM in mice, TNF-α and IFN-γ, as well as others, are known to be increased in human pancreas exocrine inflammation, which we had observed in our patients prior to the diagnosis of CPI-DM (52). In order to model the islet cell environment in CPI-DM with human β cells, we investigated human β cell responses to inflammatory mediators. We focused on the effects of IFN-γ because of our findings in the mouse model, and in preliminary studies we found that the levels of IFN-γ, more so than other inflammatory cytokines, were enhanced 4.05- ± 0.53-fold in cultures of islet cells with allogeneic PBMCs when anti–PD-1 mAb was added. We performed scRNA-Seq of islet cells that were cultured with IFN-γ (Supplemental Table 5). The effects of IFN-γ were greatest on β cells, reflected by the IFN-γ likelihood with culture, analyzed by MELD (Figure 6, A and B), though there were effects on other islet cells (e.g., GCG^+^ α cells and SST^+^ δ cells). These analyses distinguished the responses of β cells to IFN-γ with a distinct β cell cluster identified with IFN-γ treatment (Figure 6C). Spectral frequency analysis with MELD identified transitional populations in β cells that were accompanied by a change from low to high *CD274* in response to IFN-γ (Figure 6, D and E). These changes in gene transcription were supported by flow cytometry (relative fold induction compared with control treatment of 2.97 ± 0.30; range 1.49 to 5.61) (Supplemental Figure 7A). TNF-α had a synergistic effect on IFN-γ induction of PD-L1 with a mean 5.38- ± 1.03-fold induction compared with control treatment and a 2.04- ± 0.21-fold induction compared with IFN-γ only (Supplemental Figure 7A). These results are consistent with previous data suggesting IFN-γ is a principal inducer of PD-L1 on β cells (48, 50) with a synergistic effect of TNF-α on PD-L1 expression. A strong correlation was observed between *CD274* and *STAT1* and *IRF1* in our RNA-Seq analysis (Supplemental Figure 7B) consistent with the known role of IRF1/STAT1 in PD-L1 regulation (51). Moreover, PD-L1 expression was reduced when we inhibited STAT1 signaling in islet cells with ruxolitinib, a selective JAK1/2 inhibitor, when islets were cultured with IFN-γ (Supplemental Figure 7C).

IFN-γ increased gene expression in pathways of cytokine production, antigen processing and presentation, and cell death (Figure 6, F and G). Select genes of interest were confirmed by qPCR (Supplemental Figure 8). Our observations in vitro with human β cells cultured with IFN-γ corresponded to our findings in PD-L1–induced diabetes in mice: we found significant overlap in genes by rank-sum test (Spearman *r* = 0.655, *P* = 0.0001 for the top 100 genes and Spearman *r* = 0.625, *P* = 3.55 × 10^–18^ for the top 1000 genes) (Figure 6H). Overlapping gene pathways included those involved in IFN-γ response, antigen processing and presentation, and cell death.

**Figure 7:**
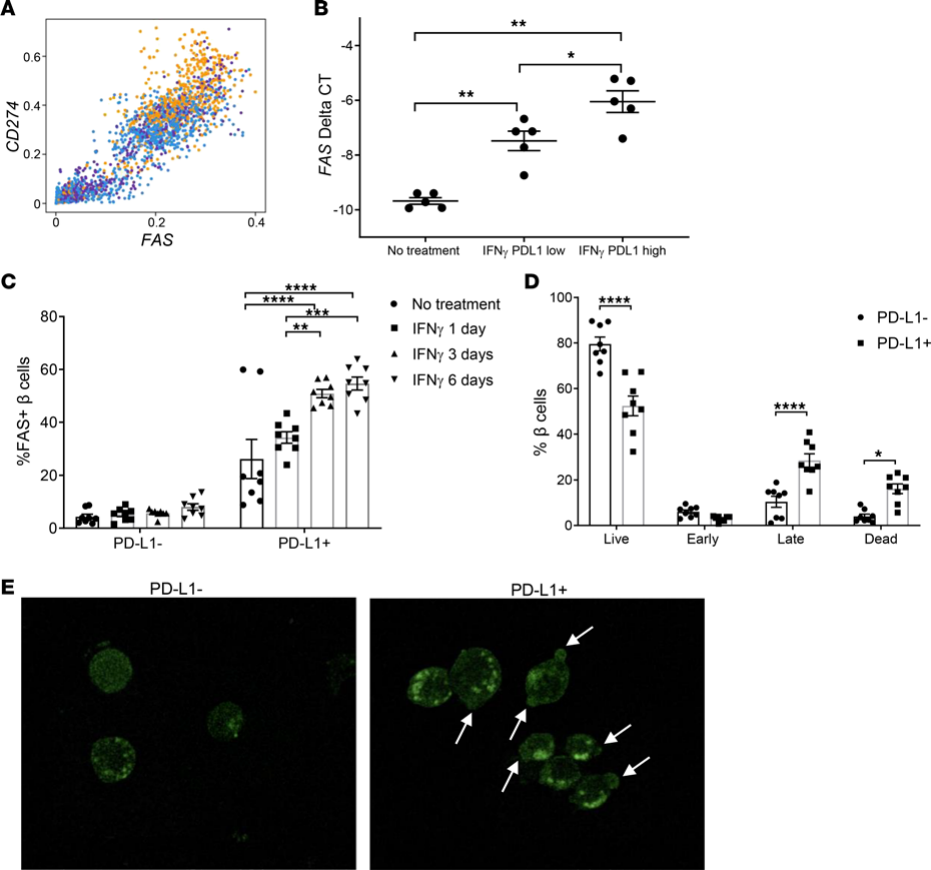
Increased FAS expression and apoptosis in PD-L1–positive β cells in response to IFN-γ. (**A**) Expression of *FAS* was highly correlated with *CD274* expression in β cells by MELD. *R*^2^ = 0.84. Individual donor cells are shown in colors. (**B**) qPCR analysis demonstrated higher expression of *FAS* in FACS-sorted β cells with higher PD-L1 expression in the presence of IFN-γ. *n* = 5 for control and culture with IFN-γ. *P ≤ 0.05, **P ≤ 0.01 by 1-way ANOVA. (**C**) FAS protein expression was increased in PD-L1^+^ β cells and increased over time in culture with IFN-γ. Two-way ANOVA. (*n* = 4, duplicate wells.) (**D**) IFN-γ induced a significant reduction in live PD-L1^+^ β cells and a significant increase in PD-L1^+^ late apoptotic and dead β cells after 6 days in culture. (*P < 0.05, **P < 0.01, ***P < 0.001, ****P < 0.0001. Two-way ANOVA with Tukey’s multiple comparison test.) (*n* = 4, duplicate wells.) Data for **B**–**D** shown as mean ± SEM. (**E**) FluoZin-3–stained β cells showing that PD-L1^+^ cells in the setting of IFN-γ treatment exhibited apoptotic blebs (white arrows). Acquired with a 63×/1.3 immersion objective lens and 10× magnification using a Leica SP8 STED 3× super-resolution microscope.

*FAS*, which encodes a cell surface death-inducing receptor, was induced in β cells by IFN-γ in our RNA-Seq analysis, and *FAS* expression and *CD274* expression were highly correlated (Figure 7, A and B). FAS was demonstrated to be more highly expressed in PD-L1–positive cells at a protein level and to increase over time with treatment (Figure 7C). After 6 days in culture with IFN-γ, we assessed apoptosis and found a significant reduction in live PD-L1–positive cells and a significant increase in PD-L1–positive late apoptotic and dead cells by flow cytometry and cell morphology (Figure 7, D and E). The higher frequency of apoptotic PD-L1–positive β cells and higher expression of FAS in these cells suggests that PD-L1 may be a marker of fragile and dying cells.

### Neutralizing IFN-γ and TNF-α in vivo in NOD mice prevents development of diabetes.

**Figure 8:**
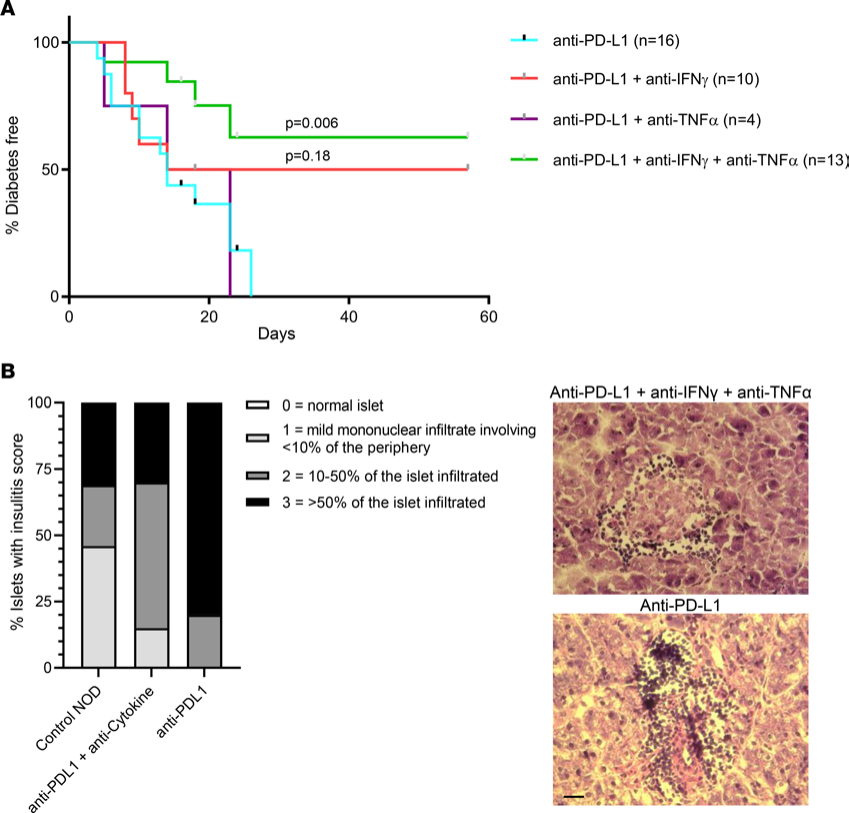
Blocking IFN-γ and TNF-α significantly delays the development of diabetes. (**A**) IFN-γ and TNF-α were neutralized in NOD mice concurrently treated with anti–PD-L1 to assess the effect on diabetes induction. Anti–TNF-α alone did not delay the induction of anti–PD-L1–induced diabetes (median time = 18.5 versus 14 days, *P* = 0.97), but neutralization of anti–IFN-γ alone did delay the time to diabetes (median time = 35.5 days, *P* = 0.18). Blocking both IFN-γ and TNF-α significantly delayed the development of diabetes (median time = undef, *P* = 0.006). *n* = 16 for anti–PD-L1 only, *n* = 10 for anti–PD-L1 + anti–IFN-γ, *n* = 4 for anti–PD-L1 + anti–TNF-α, *n* = 13 for anti–PD-L1 + anti–IFN-γ + anti–TNF-α. χ^2^, log-rank (Mantel-Cox) test. (**B**) Grades of insulitis observed in age-matched control NOD (*n* = 3 mice), anti–PD-L1 only (*n* = 3 mice), and anti–PD-L1 + anti–IFN-γ + anti–TNF-α–treated NOD mice (*n* = 4 mice). *n* = 10–20 islets per condition. A greater degree of insulitis was observed with anti–PD-L1 only (χ^2^ *P* < 0.0001). Representative H&E images are shown for anti–PD-L1 only and anti–PD-L1 + anti–IFN-γ + anti–TNF-α islets. Scale bar: 25 μm.

Our data from the murine model and with human islets identified IFN-γ and TNF as key mediators of CPI-DM. To test whether CPI-DM may be preventable by neutralizing IFN-γ and TNF, we treated young NOD mice with anti–IFN-γ and anti–TNF-α and compared the time to diabetes with anti–PD-L1 treatment. Anti–TNF-α alone did not delay the induction of anti–PD-L1–induced diabetes (median time = 18.5 days versus 14 days, *P* = 0.97), but neutralization of anti–IFN-γ alone trended toward a delay in the time to diabetes (median time = 35.5 days versus 14 days, *P* = 0.18). Blocking both IFN-γ and TNF-α significantly delayed the development of diabetes (median time = undef versus 14 days, *P* = 0.006) (Figure 8A). We observed a greater degree of insulitis in anti–PD-L1–treated mice compared with control NOD and mice treated with anti–IFN-γ with anti–TNF-α, suggesting that cytokine blockade may have an impact on the degree of immune cell islet infiltrate (Figure 8B).

## Discussion

Despite the well-documented occurrence of fulminant diabetes following the use of CPIs, the mechanisms that lead to this irAE are not clear. CPI-DM occurs more frequently after PD-1/PD-L1 blockade alone or in combination with anti–CTLA-4 mAb but not after CTLA-4 blockade alone. Using clinical studies and model systems, we have addressed the cellular and molecular mechanisms that are involved in this irAE. In patients with CPI-DM, we found reduced pancreatic volume by CT scan and increased pancreatic enzymes in serum, leading us to hypothesize that inflammation within the pancreas played a role in the pathogenesis. We identified changes in both β cells and immune cells following CPI and differential effects of anti–CTLA-4 and anti–PD-L1 mAbs that may account for the clinical differences in diabetes with these CPIs. Our findings indicate that anti–PD-L1 blockade activates T cells, including cytolytic CD8^+^ T cells, to produce cytokines that result in β cell death. In the setting of CPI-DM in mice, IFN-γ–activated pathways are expressed in β cells, and cytokines that can directly cause cell death or recruit other cells are produced by the endocrine cells. Our findings also show transcriptional changes in a subset of β cells consistent with dedifferentiation similar to our observations of β cells in NOD mice with spontaneous diabetes and other model settings (49, 54). Altogether, our data suggest that PD-1/PD-L1 blockade leads to inflammation in the pancreas that initiates a feed-forward cycle in which immune and target cells participate in the loss of β cells. Interrupting this cycle with antibodies to neutralize inflammatory mediators can prevent disease.

The increased levels of amylase and lipase suggesting pancreatic inflammation prior to the onset of CPI-DM may represent the initiation of the pathologic process that then leads to reduced pancreatic volume. Others have noted evidence of pancreatic inflammation, including shrinkage of pancreatic volume with fulminant CPI-DM, but there is limited histology from these cases or comparisons to those without CPI-DM (55–57). Interestingly, in patients with T1D, pancreatic volume may also decline, which suggests a common initiating mechanism with spontaneous disease (58). Not all CPI-treated patients who developed DM had increased levels of amylase and lipase, possibly because of the infrequency of monitoring. Curiously, in our experience the pancreatic enzyme elevations are generally silent clinically and, in most cases, do not lead to exocrine insufficiency. Hence, monitoring these chemistries may indicate impending endocrine damage.

A primary role for cytotoxic CD8^+^ T cells in the pathogenesis of CPI-DM is suggested by finding CD8^+^ cells infiltrating the islets in a patient who succumbed with CPI-DM and in NOD mice with PD-L1–induced diabetes, consistent with the known role of PD-1 signaling in limiting the expansion of CD8^+^ T cells (59). *Fasl* was elevated in anti–PD-L1–treated mouse islet-infiltrating CD8^+^ T cells compared with anti–CTLA-4 as well as other cytotoxic effector molecules such as *Gzma*, *Gzmb*, and *Prf1*. Perforin and granzyme B may have a shared role in tumor killing but also β cell death in T1D (60–65). These same transcriptional findings were not seen with anti–CTLA-4 mAb, suggesting a critical mechanistic difference. We also found differences between macrophages in the islets of anti–CTLA-4– and anti–PD-L1–treated mice. The differences were in pathways involved in recruitment of immune cells and cellular infiltration (e.g., *Cxcl9* and *Cxcl10*) but also in the production of mediators and ligands that may be directly toxic to β cells, including *Fas* and *Tnfsf10* as has been reported previously (53).

Importantly, our scRNA-Seq studies suggest that β cells contribute to the progression of CPI-DM. PD-L1 is expressed on β cells that have been exposed to IFN-γ. The chemokine *Cxcl10*, which has been shown to recruit pathologic T cells to inflamed islets, was increased in β cells from anti–PD-L1–treated mice, most likely in response to IFN-γ signaling (66). Importantly, qPCR analysis of pancreatic tissue from our patient with CPI-DM showed a 16-fold elevation in *CXCL9* when compared with pancreatic tissue from control individuals (ΔCt –6.94 for CPI-DM versus median ΔCt –11.18 for normal), consistent with the scRNA-seq data from the CPI-treated mice. Thus, in response to inflammatory cytokines including IFN-γ, islet cells may secrete chemokines capable of recruiting and activating immune cells.

We describe mechanisms that β cells use for protection but ultimately result in their loss. PD-L1 and IDO1 were increased on β cells that were exposed to inflammatory cytokines. However, expression of these ligands alone was insufficient to protect β cells, as we also found that expression of PD-L1 was associated with mediators of cell death, such as FAS, and increased β cell killing in vitro. In mice we identified a subpopulation of β cells with features suggesting dedifferentiation, analogous to our findings of dedifferentiated β cells that develop during the progression of spontaneous diabetes in NOD mice and in streptozotocin-treated mice (49, 54). The gene signatures of dedifferentiation may lead to β cell dropout and loss of functional β cell mass (67). This mechanism, together with direct β cell killing, may account for the rapid kinetics of diabetes in the mice and patients.

Our studies with CPI-DM in mice suggest that antiinflammatory drugs may be considered for secondary prevention/reversal of CPI-DM. When administered with anti–PD-L1 mAb, neutralizing both IFN-γ and TNF-α in NOD mice treated with anti–PD-L1 inhibited the development of CPI-DM. Further understanding the mechanisms of glycemic deterioration in patients with CPI-DM is important, as some patients may have residual C-peptide and may benefit from therapeutic intervention to preserve remaining β cells. TNF-α blockade has been used to treat certain irAEs resulting from CPIs, such as colitis, which shares activation of TNF and IFN-γ pathways (68, 69). Importantly, there does not seem to be a negative impact on tumor responses or survival in patients treated with infliximab (70, 71). Moreover, a JAK inhibitor, which can block IFN-γ signaling, was used to treat a patient with CPI-induced colitis (68). However, further investigation would be needed in mice and humans regarding the impact and timing of inhibiting these and other inflammatory mediators on antitumor responses.

There are limitations to our studies. Studies of pancreatic tissue from patients who have CPI-DM are extremely rare, and our studies were limited to a single patient who died after the onset of this irAE. In our studies we used anti–PD-L1 rather than anti–PD-1 mAb in order to avoid cell depletion, which is a known effect of the latter and would complicate our mechanistic studies (72). However, we cannot exclude that there was antibody-dependent phagocytosis mediated by the anti–PD-L1 mAb or possibly nonspecific activation of immune cells by engagement of Fc receptors by anti–PD-L1 mAb that may have contributed to the findings. In addition, by using inbred NOD mice, we are unable to address the role of the genetic background on development of diabetes after CPI, which we and others found to be significant (8, 11, 24). Whether the effects of cytokines on β cell killing and dedifferentiation are direct or indirect by regulating immune cells is not certain. A role for β cell antigen–reactive T cells in CPI-DM remains unknown and warrants further investigation, but with class I MHC tetramers (HLA-A2 or K^d^), we have not identified expansion of CD8^+^ T cells reactive to conventional diabetes antigens in humans or NOD mice with CPI-DM. CPI-DM is restricted to NOD mice, but these observations suggest that the mechanisms of CPI-DM differ from spontaneous T1D and may involve potentially novel targets. Finally, the NOD model has limitations including the sex bias for diabetes, and our mice do not harbor tumors; the effects of the antitumor response on diabetes and vice versa could not be addressed.

In summary, our studies identify mechanisms that lead to CPI-DM in humans and in a preclinical mouse model. Diagnostic or therapeutic targets or even strategies to protect β cells from the effects of inflammatory mediators of checkpoint inhibition without impacting tumor responses are needed. Our findings may not only identify potential avenues for treatment but also identify mechanisms that are active in spontaneous T1D and can be targeted.

## Methods

### Assessment of pancreatic changes in CPI-treated patients.

Patients were identified through the Endocrinology and Oncology services at Yale New Haven Hospital. Patients who met criteria for CPI-DM were previously described (8). Briefly, they a) had new-onset hyperglycemia requiring exogenous insulin treatment without a prior history of diabetes or with a history of type 2 diabetes and were on oral medications and became insulin dependent or showed worsening control on insulin and b) exhibited continued insulin requirements for more than 1 month and had evidence of insulin deficiency by presenting with diabetic ketoacidosis or low or absent random C-peptide. CPI-treated control nondiabetic patients were identified in collaboration with the Oncology service as patients treated with similar CPIs who did not develop diabetes within a similar time frame after starting CPI therapy. For analysis of pancreatic volumes and pancreatic enzyme changes, data were pulled from patient electronic health records (Epic; Epic Systems). Clinical characteristics of patients in these studies are summarized in Supplemental Table 1.

Pancreatic volumes were assessed by comparing pretreatment and posttreatment imaging. To determine pancreatic volumes, for each image in which the pancreas was present, a manually defined region of interest around the pancreas was created, giving the area of the pancreas on a specified image. This was multiplied by the imaging slice thickness to calculate the volume of pancreatic tissue on the image. These volumes were summated to compute the total pancreatic volume.

### Tissues and immunohistochemistry.

Paraffin-embedded human pancreatic tissue was obtained from the Yale Pathology Department. Samples consisted of 3 specimens of normal pancreatic tissue, 2 specimens of autoimmune pancreatitis, 3 specimens of chronic pancreatitis, and 1 specimen from a person with CPI-DM. Immunofluorescence staining was performed on tissue sections that were deparaffinized using xylenes and then immersed in graded alcohols. Slides were subjected to antigen retrieval using a citrate-based solution with 0.05% Tween 20 (BD antigen unmasking solution) at 100°C for 30 minutes. Slides were allowed to cool and washed 3× in PBS for 5 minutes each time. Sections were permeabilized using PBS/0.2% Triton X-100 for 30 minutes and then blocked with 5% normal goat sera in PBS for 1 hour at room temperature (RT). Primary antibodies were diluted in 5% normal goat sera at the appropriate dilution overnight at 4°C. The following morning secondary antibodies were incubated for 1 hour at RT. Following staining slides were washed, dried, and coverslipped with ProLong Gold Antifade with DAPI (Invitrogen). Antibodies used for staining were mouse anti–PD-L1 (BioLegend, 29E.2A3, catalog 329702, 1:100 dilution), mouse anti-IDO1 (Abcam, 4D2, catalog ab55305, 1:100 dilution), guinea pig anti-insulin (Invitrogen, catalog 180067, 1:300 dilution), and rabbit anti-CD45 (Abcam, catalog ab10558, 1:100 dilution). Secondary antibodies were goat anti-rabbit Alexa Fluor 647 (Invitrogen, catalog A-21245), Alexa Fluor 488 goat anti-guinea IgG (Invitrogen, catalog A-11073), and Rhodamine Red X–conjugated AffiniPure donkey anti-mouse (Jackson ImmunoResearch, catalog 715-295-150), all used at a 1:200 dilution. Immunofluorescence was visualized using a Zeiss Axiovert 200M fluorescence microscope. Confocal images were taken with a Zeiss LSM 880 with Airyscan microscope.

For multispectral staining of the human autopsy pancreas, archival paraffin-embedded pancreatic tissue was sectioned at 5 mm thickness and stained using Opal reagents (Akoya Biosciences) following the manufacturer’s standard protocol. The multiplex panel included DAPI for nuclear counterstaining, CD4 (Abcam, EPR6855, catalog ab133616, 1:150 dilution), CD8 (Leica, 4B11, catalog CD8-4B11-L-CE, 1:600 dilution), and chromogranin A (Abcam, catalog ab45179, 1:300 dilution). Single controls and an unstained slide were stained with each group of slides. After staining, the sections were mounted in VECTASHIELD HardSet mounting media (Vector Laboratories) and stored at 4°C for up to 48 hours prior to image acquisition. Multispectral imaging and acquisition at 20× original magnification (numerical aperture 0.75) was performed using the integrated Vectra 3 automated quantitative pathology imaging system (PerkinElmer) as previously described (73). Images were analyzed using inForm software (PerkinElmer/Akoya Biosciences).

For immunohistochemistry staining of human and mouse paraffin-embedded pancreatic tissue, antigen retrieval was done with citrate buffer pH 6.0. Endogenous peroxidase was quenched with 3% hydrogen peroxide. Primary antibodies were rabbit anti–IFN-γ (Novus, JM10-10, catalog NBP2-66900, 1:75 dilution), rabbit anti–TNF-α (Novus, catalog NBP1-19532, 1:200 dilution), goat anti-mouse Cxcl10 (Novus, catalog AF466-NA, 10 μg/mL), rat anti-mouse CD45 (Abcam, I3/2.3, catalog ab25386, 1:100 dilution), mouse anti-human CD45 (Dako, 2B11+PD7/26, catalog M0701, 1:100 dilution), and guinea pig anti-insulin (Thermo Fisher Scientific, catalog GPASW-INS-7S, 1:80 dilution). Secondary detection of these antibodies was accomplished using horseradish peroxidase–conjugated secondary antibodies: goat anti-rabbit Mach 2 (Biocare Medical, catalog RHRP520, also cross-reacts and used with guinea pig), rabbit anti-goat (Jackson ImmunoResearch, catalog 305-035-045), goat anti-mouse Mach 2 (Biocare Medical, catalog MHRP520), and rabbit anti-rat (Jackson ImmunoResearch, catalog 312-035-048). The slides were visualized using diaminobenzidine tetrahydrochloride (Biocare Medical).

Immunofluorescence staining of mouse pancreas frozen sections was done after fixing with acetone and blocking with 5% donkey serum in 0.25% Triton X-100 for 1 hour. Primary antibody staining was done at 4°C overnight in 1% BSA/0.25% Triton X-100. Secondary antibody staining was done for 1 hour at RT in 1% BSA/0.25% Triton X-100. Samples were mounted in ProLong Gold antifade reagent with DAPI (Invitrogen). Primary antibodies were guinea pig anti-insulin (Invitrogen, catalog 180067, 1:400 dilution) and rat anti-mouse CD45 (BioLegend, 30-F11, catalog 103102, 1:400 dilution). Secondary antibodies were Alexa Fluor 488 goat anti-guinea IgG (Invitrogen, catalog A-11073, 1:200 dilution) and Rhodamine Red X donkey anti-rat (Jackson ImmunoResearch, catalog712-295-153, 1:200 dilution).

### Human islet cultures and cytokine treatment.

Human islets were obtained from adult, nondiabetic organ donors from Prodo Laboratories, Inc., or the Integrated Islet Distribution Program at City of Hope. Islets were cultured in CMRL 1066 medium (Gibco) supplemented with 10% FBS (MilliporeSigma), 10 mM HEPES (AmericanBio), 2 mM l-glutamine (MilliporeSigma), and 1% pen-strep (Gibco). Where indicated, islets were treated with 25 or 100 ng/mL IFN-γ (R&D Systems) or 10 ng/mL TNF-α (R&D Systems). In some experiments, islets were pretreated with 5 μM ruxolitinib (Selleckchem) for 1 hour prior to the addition of IFN-γ. Islets were harvested at indicated time points and dissociated into single-cell suspensions using 0.05% trypsin-EDTA (Gibco). Cells were stained with FluoZin-3 (Invitrogen) and TMRE (Life Technologies) for β cell isolation experiments and sorted using a FACSAria II (BD).

### Flow cytometry and β cell apoptosis analysis.

Cultured human islets were dissociated into single-cell suspensions and stained with LIVE/DEAD Fixable Yellow Dead Cell Stain (Invitrogen) followed by FluoZin-3 (Invitrogen). Cells were then blocked with Human Fc Block (BD Biosciences) and stained with surface antibody: PE-CY7 anti–PD-L1 (BioLegend, 29E.2A3, catalog 329718) or BV421 anti–PD-L1 (BioLegend, 29E.2A3, catalog 329714).

For apoptosis and FAS expression analysis, islets were harvested, dispersed into single cells, and stained with PE anti-CD45 (BD Pharmingen, HI30, catalog 555483) (to exclude immune cells from analysis), APC anti-FAS (BioLegend, DX2, catalog 305612), and PE-CY7 anti–PD-L1 (BioLegend, 29E.2A3, catalog 329718) followed by 7-aminoactinomycin D (BioLegend) and PB annexin V (BioLegend) per company protocol. Cells were analyzed with LSRFortessa (BD) and analyzed with FlowJo (version 9).

### Studies in NOD mice with CPI-induced diabetes.

Six-week-old female NOD mice (NOD/ShiLtJ) were purchased from The Jackson Laboratory and maintained in our facility under specific pathogen–free conditions. Mice were treated at approximately 7 weeks with 100 μg anti–PD-L1 (Bio X Cell, clone 10F.9G2, catalog BP0101 or BE0101) or anti–CTLA-4 (Bio X Cell, clone 9D9, catalog BP0164 or BE0164) every 3 days. Mice were monitored for the development of hyperglycemia every 3 days with diabetes defined as blood glucose level more than 250 mg/dL on 2 occasions. Mice were sacrificed upon developing diabetes. For anticytokine studies mice were treated with anti–PD-L1 alone or with 250 μg or 500 μg of anti–IFN-γ (Bio X Cell, clone XMG1.2, catalog BP0055) and/or 250 μg or 500 μg of anti–TNF-α (Bio X Cell, clone XT3.11, catalog BP0058). Mice received a total of 4 to 6 doses of antibody treatment rounds per experiment.

Insulitis was assessed using ImageJ (NIH) with a 4-point scoring system: 0 = normal islet, 1 = mild mononuclear infiltrate involving <10% of the periphery, 2 = 10% to 50% of the islet infiltrated, 3 = >50% of the islet infiltrated.

### Bulk RNA-Seq of CPI-treated NOD mouse islets.

Approximately 7-week-old NOD mice were treated with anti–PD-L1 or anti–CTLA-4 every 3 days for 2 doses. Two days after the second dose mice were sacrificed and islets were harvested and processed into single cells. For NOD controls, mice were sacrificed at 11 weeks old and processed similarly to antibody-treated mice. Cells were stained with TMRE (Life Technologies), FluoZin-3 (Invitrogen), and BV421 anti-mouse CD45 (BioLegend, 30-F11, catalog 103133). Live CD45^+^ and live CD45^–^ cells were sorted using a FACSAria II (BD), cells were frozen in RLT buffer, and subsequent RNA isolation was performed using QIAGEN RNeasy Plus Micro Kit. RNA was subsequently processed at the Yale Center for Genome Analysis (YCGA). Briefly, the SMARTer universal low-input RNA kit (Takara Bio Inc) was used to convert RNA to cDNA. Bioanalyzer 2100 was used to measure cDNA quality and concentration. Nextera DNA Library Preparation Kit was used to make libraries, and quality control of the libraries was performed on an Agilent Technologies 2100 Bioanalyzer using an Agilent high-sensitivity chip. Multiplexed DNA libraries were normalized to 10 nM and then pooled in equal volumes with each unique barcoded sample. The purified DNA was captured on an Illumina flow cell for cluster generation. Libraries were sequenced on the HiSeq 4000 (Illumina) following the manufacturer’s protocols. Data were analyzed using Partek software.

### scRNA-Seq and analysis.

scRNA-Seq was performed on human β cells from 3 islet donors cultured for 24 hours with and without human IFN-γ (25 ng/mL). Characteristics of the 3 islet donors are shown in Supplemental Table 5. The islets were dissociated into single cells, stained with FluoZin-3 and TMRE, sorted, and processed by 10x Genomics at the YCGA. Cell viability was assessed using the Countess II Automated Cell Counter (Life Technologies).

For mouse single-cell experiments, approximately 7-week-old NOD mice were treated with anti–PD-L1 or anti–CTLA-4 as described previously every 3 days for 2 doses. Two days after the second dose, mice were sacrificed, and islets were harvested and processed into single cells. Samples from *n* = 3 mice treated with anti–PD-L1 were combined, and samples from *n* = 3 mice treated with anti–CTLA-4 were combined. Cell viability was assessed using the TC20 Automated Cell Counter (Bio-Rad).

Single-cell suspension in RT Master Mix was loaded on the Single Cell Chip (Chromium Next GEM reagents from 10x Genomics) and partitioned with a pool of about 750,000 barcoded gel beads to form nanoliter-scale gel beads-in-emulsions (GEMs). Upon dissolution of the gel beads in a GEM, the primers with the unique cell barcodes were released and mixed with cell lysate and Master Mix. Incubation of the GEMs then produced barcoded, full-length cDNA from poly-adenylated mRNA. Silane magnetic beads (10x Genomics) were used to remove leftover biochemical reagents and primers from the post-GEM reaction mixture. Full-length, barcoded cDNA was then amplified by PCR to generate sufficient mass for library construction. Enzymatic fragmentation and size selection were then used to optimize the cDNA amplicon size prior to library construction. The final libraries contained the P5 and P7 primers used in Illumina bridge amplification. Analysis steps, such as demultiplexing, alignment, and gene counting, and visualization to generate expression data with single-cell resolution, were performed.

PHATE, Multiscale PHATE, and MELD algorithms were used to analyze RNA-Seq data. PHATE is an embedding tool that highlights local and global structure of the data and identifies transitions and progressions in the data (74). Multiscale PHATE is another embedding tool that allows for interactivity with local and global structures in data. Based on the same principles as PHATE, Multiscale PHATE is able to visualize the coarse-grained structure of data at high levels to create summarizations and then zoom in to smaller cell types without losing information (75). In this manuscript, Multiscale PHATE was used purely as a visualization tool to illustrate cellular populations across granularities. MELD is a single-cell compositional analysis method that quantifies the likelihood of a cellular state appearing in either the control or perturbation condition for every cell in the cellular manifold (76). By tracking how gene expression and cell abundance change with this likelihood, we can understand how the perturbation affects cell state. Before the PHATE and MELD algorithms were applied, preprocessing steps for the scRNA-Seq data were performed to reduce their noise (77). Specifically, dead cells were identified by their high mitochondrial gene expression levels and removed. Also, cells were filtered by library size, where cells with library sizes that were significantly smaller or larger than average were removed, as these constitute empty droplets and doublets, respectively. Last, genes that were expressed in relatively few cells were removed because there was not enough information to make solid inferences on their expression. The cells were then normalized by library size so that expression levels could be properly compared between cells that started off with varying numbers of mRNA molecules. Since some genes are orders of magnitude more common than others, a square-root transform was applied so that the high-expression genes did not dominate the variation. To identify cell populations of pancreatic islet and immune cells, Leiden clustering was performed (78). The marker genes, or gene signatures, of each cluster were used to annotate it with a biologically meaningful label describing the cell identity represented by the cluster. The process of identifying clusters involved referencing external sources of information that describe the expected expression profiles of individual cell identities, such as scientific literature and databases. The statistical Wilcoxon rank-sum test was used to rank genes by their difference in expression between the anti–PD-L1 and anti–CTLA-4 mouse groups (77). Pathway analysis was performed with Metascape (79) and Ingenuity Pathway Analysis (QIAGEN).

### qPCR.

RNA isolation was performed using QIAGEN RNeasy Plus Mini or Micro Kits, then converted to cDNA (High-Capacity cDNA Reverse Transcription Kit, Applied Biosystems), and quantitative real-time PCR was performed using QuantiFast SYBR Green PCR Kit (QIAGEN). Primer pairs are listed in Supplemental Table 6. The *ACTB* housekeeping gene was used for normalization, and gene transcription is presented as ΔCt = Ct *ACTB* − Ct *target gene*.

### Data availability.

Data are available at NCBI Gene Expression Omnibus at the following accession numbers: GSE209587 for the mouse bulk RNA-Seq, GSE208644 for the mouse scRNA-Seq, and GSE161465 for the human islet data.

### Statistics.

The data were analyzed with GraphPad Prism 8. Unless indicated, all data are presented as mean ± SEM. Differences with *P* < 0.05 were considered statistically significant. Survival curves were used for comparison of diabetes development in treatment groups and comparison between groups done by log-rank (Mantel-Cox) test. Statistical analysis of scRNA-Seq data is detailed above under the methodology for those analysis. Rank order analysis was done of top differentially expressed genes by lowest *P* values. Where applicable, the mouse differential expression gene lists were converted to their human counterpart using the publicly available Mouse/Human Orthology with Phenotype Annotations table. The gene lists were ranked by their respective log 2-fold changes, and their Spearman correlation coefficient with associated *P* value was calculated.

### Study approval.

The human study was approved by the institutional review board at Yale University. All animal use protocols were approved by the Yale University Institutional Animal Care and Use Committee.

## Author contributions

ALP and KCH conceptualized and designed the experiments, analyzed data, and wrote the paper. ALP conducted the experiments. SD, SPW, and NKS conducted experiments and reviewed and edited the manuscript. KCD, MK, DBB, AT, and SK analyzed RNA-Seq data, helped with data visualization, and reviewed and edited the manuscript. GI performed data analysis of CT scans and reviewed and edited the manuscript. MER, AMS, HMK, ZQ, AY, MLY, MJM, JSP, and MSA contributed to conceptualization of experiments, data analysis, and reviewing and editing of the manuscript.

## Supplementary Material

Supplemental data

## Figures and Tables

**Table 1:**
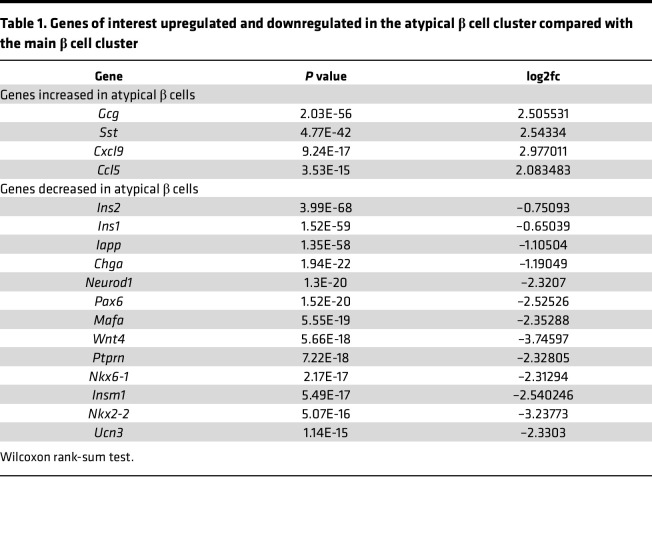
Genes of interest upregulated and downregulated in the atypical β cell cluster compared with the main β cell cluster

## Version 1 08/04/2022

In-Press Preview

## Version 2 09/08/2022

Electronic publication
