# Supplementary material for: Immune cells and their inflammatory mediators modify β cells and cause checkpoint inhibitor–induced diabetes
Source: JCI Insight. 2022 Sep 8;7(17):e156330. doi: 10.1172/jci.insight.156330 (PMC9536276; doi:10.1172/jci.insight.156330)
Supplement: Supplemental data [file jciinsight-7-156330-s029.pdf]

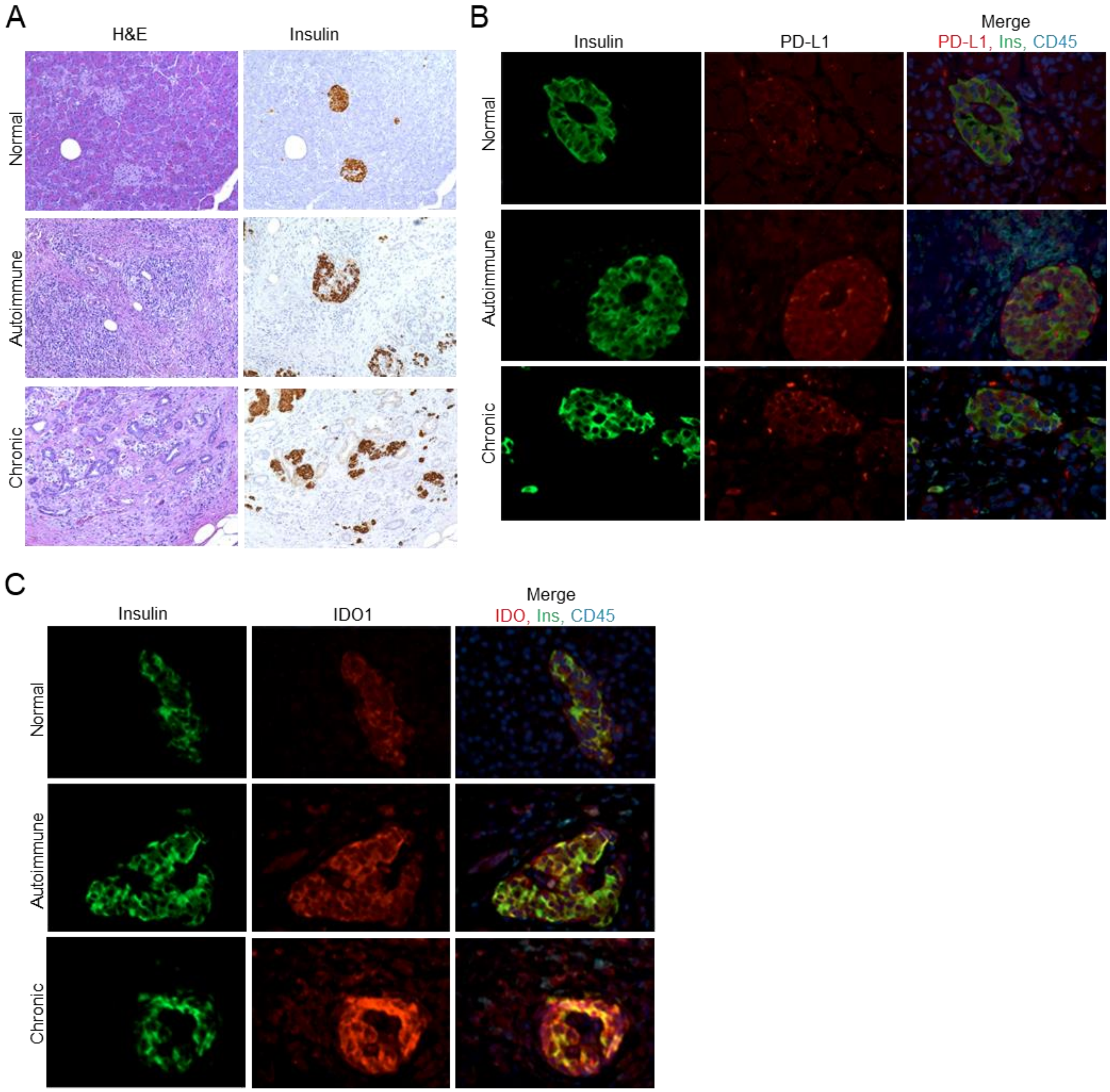

Supplemental Figure 1. PD-L1 and IDO1 are expressed in islets in the setting of pancreatitis. (A) Representative images of H&E and insulin staining from pancreatic samples with known inflammation, autoimmune and chronic pancreatitis, and normal pancreas. Taken with a 10x/25 eyepiece and 10x objective (Leica DMi8). **(B)** Representative images of immunostaining of pancreatic samples with autoimmune and chronic pancreatitis for PD-L1 (green=insulin, red = PD-L1, blue=CD45). **(C)** Representative images of immunostaining of pancreatic samples with autoimmune and chronic pancreatitis for IDO1 (green=insulin, red = IDO1, blue=CD45). Images for B and C were taken with a 10x/23 eyepiece and 20x objective (Zeiss Axiovert 200M).

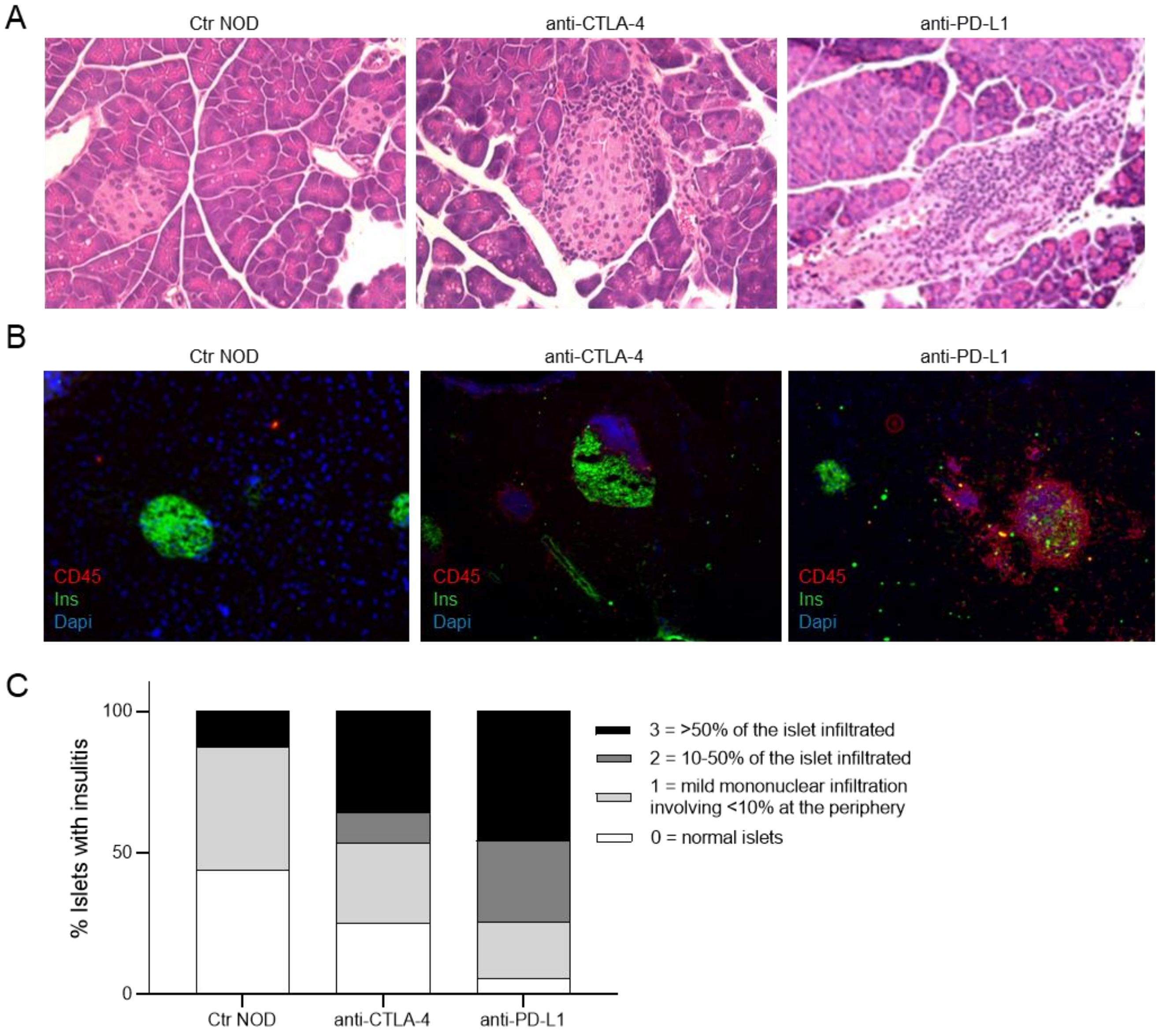

Supplemental Figure 2. Development of diabetes in anti-PD-L1 treated NOD mice. (A) H&E and **(B)** immunofluorescent staining for CD45 and Insulin show immune infiltrates in islets from mice treated with anti-CTLA-4 and anti-PD-L1. **(C)** Grades of insulitis observed in anti-PD-L1 and anti-CTLA-4 treated NOD mice. N=16 islets for control NOD, N=56 islets for anti-CTLA-4 treatment, and N=35 islets for antiPD-L1 treatment. Chi-Square $p<0.0001$. Taken with a 10x/25 eyepiece with 40x and 10x objective lens for (A) and (B) respectively (Leica DMi8).

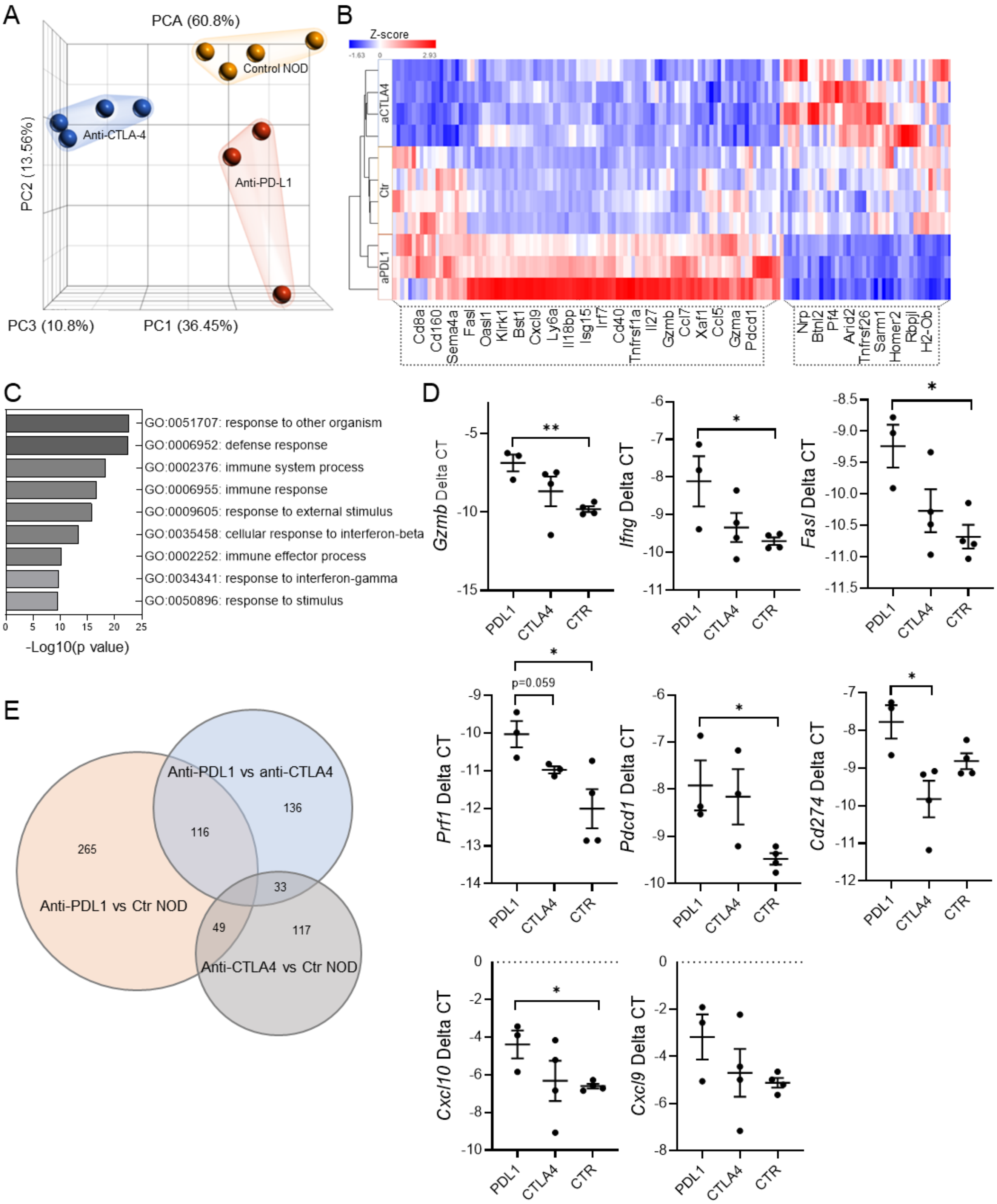

Supplemental Figure 3. Islet infiltrating CD45+ cell transcriptional changes by bulk RNA-seq in anti-PD-L1 treated NOD mice. 7-week-old NOD mice were treated with anti-PD-L1 or

anti-CTLA-4 and islets harvested prior to development of diabetes for bulk RNA sequencing to characterize differences in transcriptional changes between CPI treatments. They were compared to islets from 11-week-old NOD mice. **(A)** PCA plot showing 3 treatment groups for islet infiltrating CD45+ cells. N=4 for anti-CTLA-4 (blue), N=3 for anti-PD-L1 (red), and N=4 for Control NOD (yellow). **(B)** Heatmap of 285 genes differentially expressed between anti-CTLA-4 and anti-PD-L1 treated CD45+ cells. Differentially expressed genes were those with p value<0.05, fold change ≥2, FDR step-up <0.1. **(C)** Top pathways representing the differentially expressed genes between anti-CTLA-4 and anti-PD-L1 treated CD45+ cells. **(D)** qPCR of cDNA from CD45+ samples used in the bulk RNA-seq experiments confirms changes in select genes. Mean +/- SEM. N=3 for anti-PD-L1, N=3-4 for anti-CTLA-4, and N=4 for control NOD. *p<0.05, ** p≤0.01 by Student's t test. **(E)** Venn diagram depicting overlapping differentially expressed genes among treatment groups.

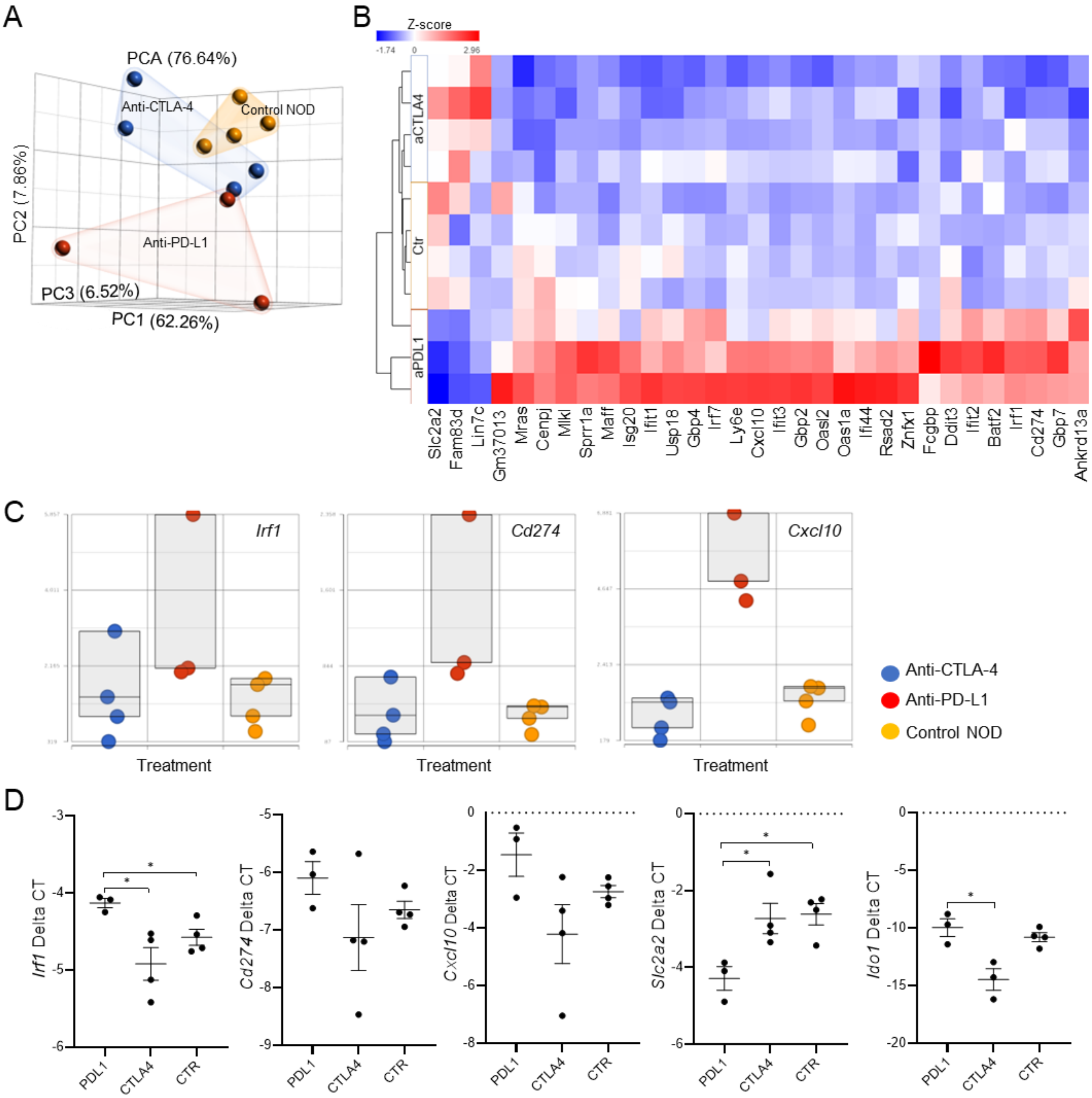

Supplemental Figure 4. Transcriptional changes in islet cells in response to checkpoint inhibition in NOD mice by bulk RNA-seq. (A) PCA plot showing 3 treatment groups for non-immune (i.e. CD45-) islet cells. N=4 for anti-CTLA-4, N=3 for anti-PD-L1, and N=4 for Control NOD. **(B)** Heatmap of 31 genes differentially expressed between anti-CTLA-4 and anti-PD-L1 treated CD45- cells. Differentially expressed genes were those with p value<0.05, fold change ≥2, FDR step-up <0.1. **(C)** Example of differentially expressed genes among the treatment groups include *Irf1*, *Cd274*, and *Cxcl10*. **(D)** qPCR of cDNA from CD45- samples confirms changes in genes identified by RNA-seq. Mean +/- SEM. N=3 for anti-PD-L1, N=3-4 for anti-CTLA-4, and N=4 for control NOD. *p<0.05 by Student's t test.

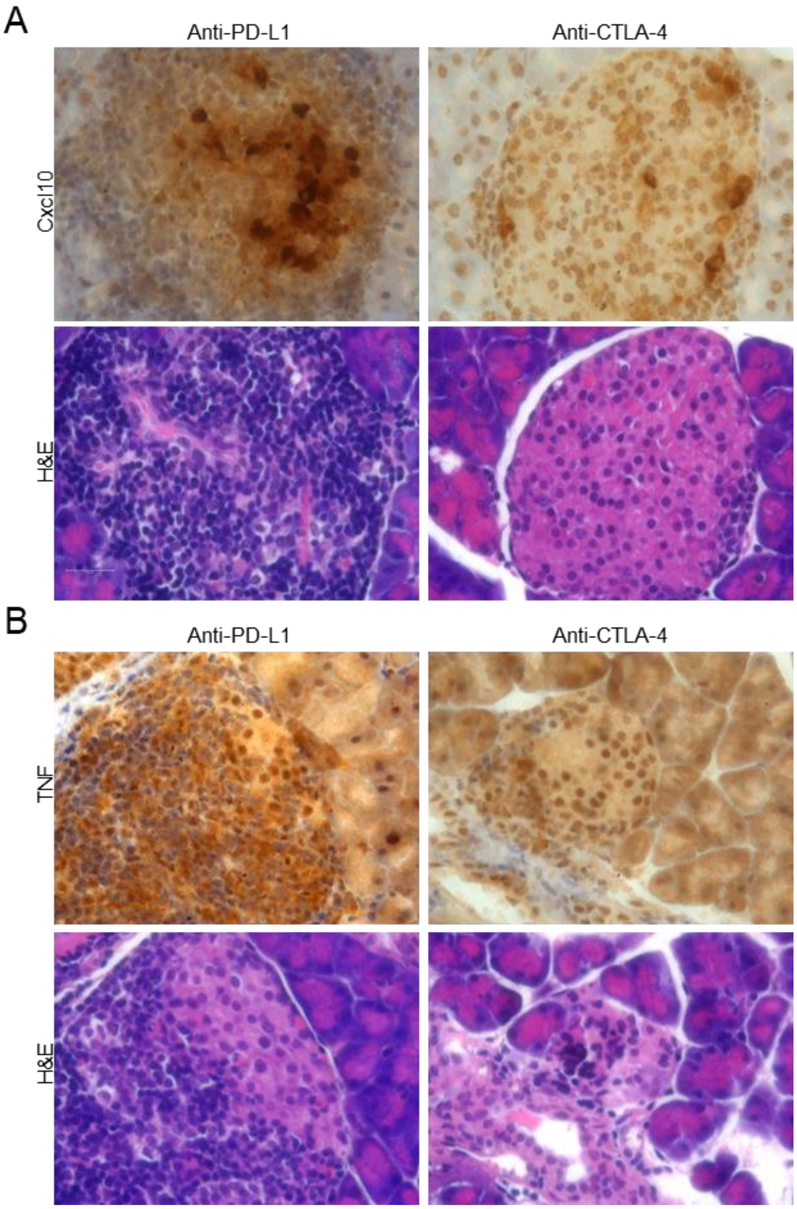

Supplemental Figure 5. Expression of inflammatory mediators in pancreatic tissue from anti-PD-L1 treated NOD mice. H&E and immunohistochemistry for **(A)** CXCL10 and **(B)** TNFα in pancreatic tissue from NOD mice following two doses of anti-PD-L1 or anti-CTLA-4 demonstrates expression of both inflammatory mediators in islet infiltrating cells of anti-PD-L1 treated mice. Scale: 25 um.

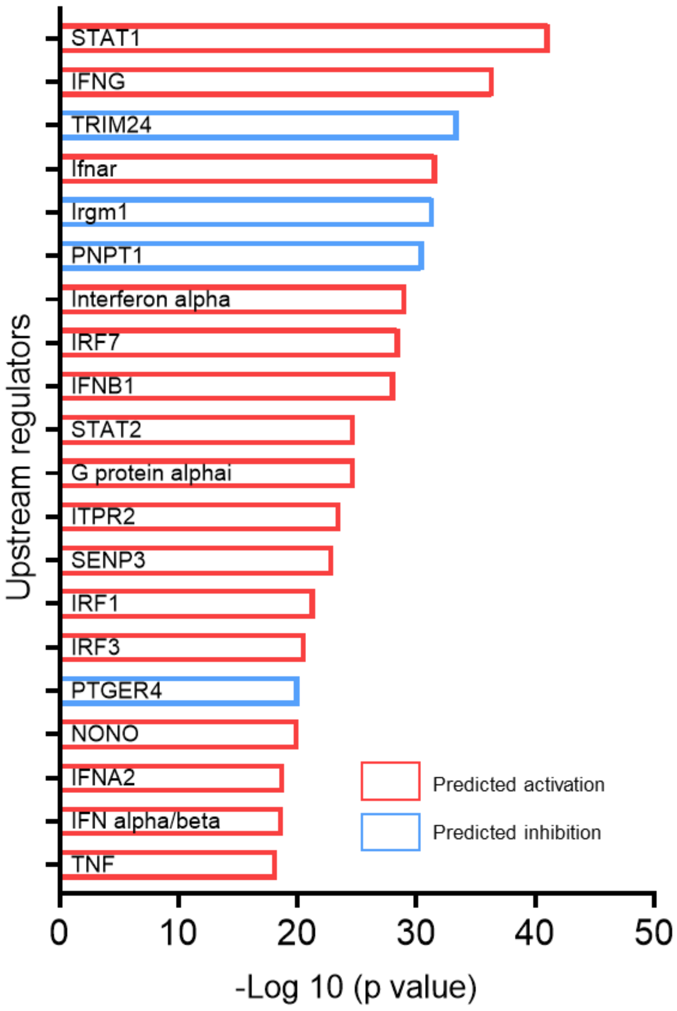

Supplemental Figure 6. Differential upstream regulators with anti-PD-L1 treatment in β cells. Top 20 predicted upstream regulators in anti-PD-L1 treated β cells compared to anti-CTLA-4 treated cells by scRNA-seq. Red=upregulated in anti-PD-L1 β cells and Blue=downregulated in anti-PD-L1 treated β cells.

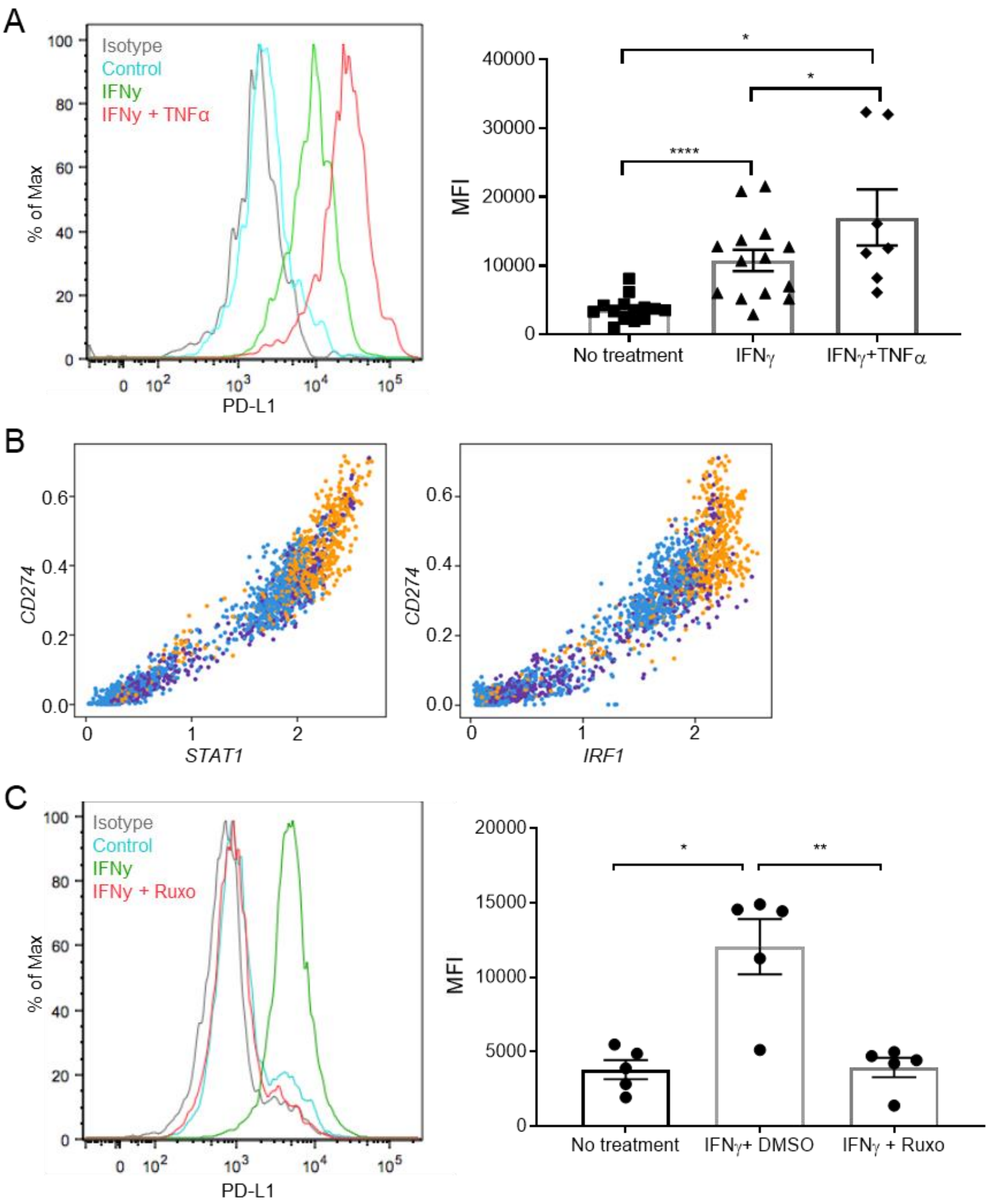

Supplemental Figure 7. Induction of checkpoint molecules in β cells in response to IFNγ. **(A)** PD-L1 expression was significantly upregulated on β cells by FACS in islets cultured with IFNγ (100ng/mL) with relative fold induction (mean (SEM)) 2.97(0.30) (range 1.49 to 5.61) vs media. TNFα (10 ng/ml) alone did not induce PD-L1 but had a synergistic effect on IFNγ induction of PD-L1 (fold induction 5.38(1.03) compared to media alone and 2.04(0.21)-fold greater than IFNγ only. N=14 for no treatment, 14 for IFNγ treatment, 7 for IFNγ+TNFα. *$p \leq 0.05$, ****$p \leq 0.0001$ by Student's t test. **(B)** MELD analysis of RNA-seq data shows a strong correlation between PD-L1 and IRF1 (R^2=0.89) and STAT1 (R^2=0.93) expression. Blue,

orange and purple symbols represent cells from each islet donor. **(C)** Inhibition of JAK1/2 with 5 μM Ruxolitinib (versus DMSO vehicle) resulted in reduced expression of PD-L1 in response to IFNγ on β cells by FACS. Mean MFI+/-SEM: 3,797+/-647.2, 12,046+/-1,852, 3,945+/-1,852 for no treatment, IFNγ+DMSO, and IFNγ+Ruxo respectively. N=5 per treatment. (One-way ANOVA, *p≤0.05, **p≤0.01 with Tukey's multiple comparison test).

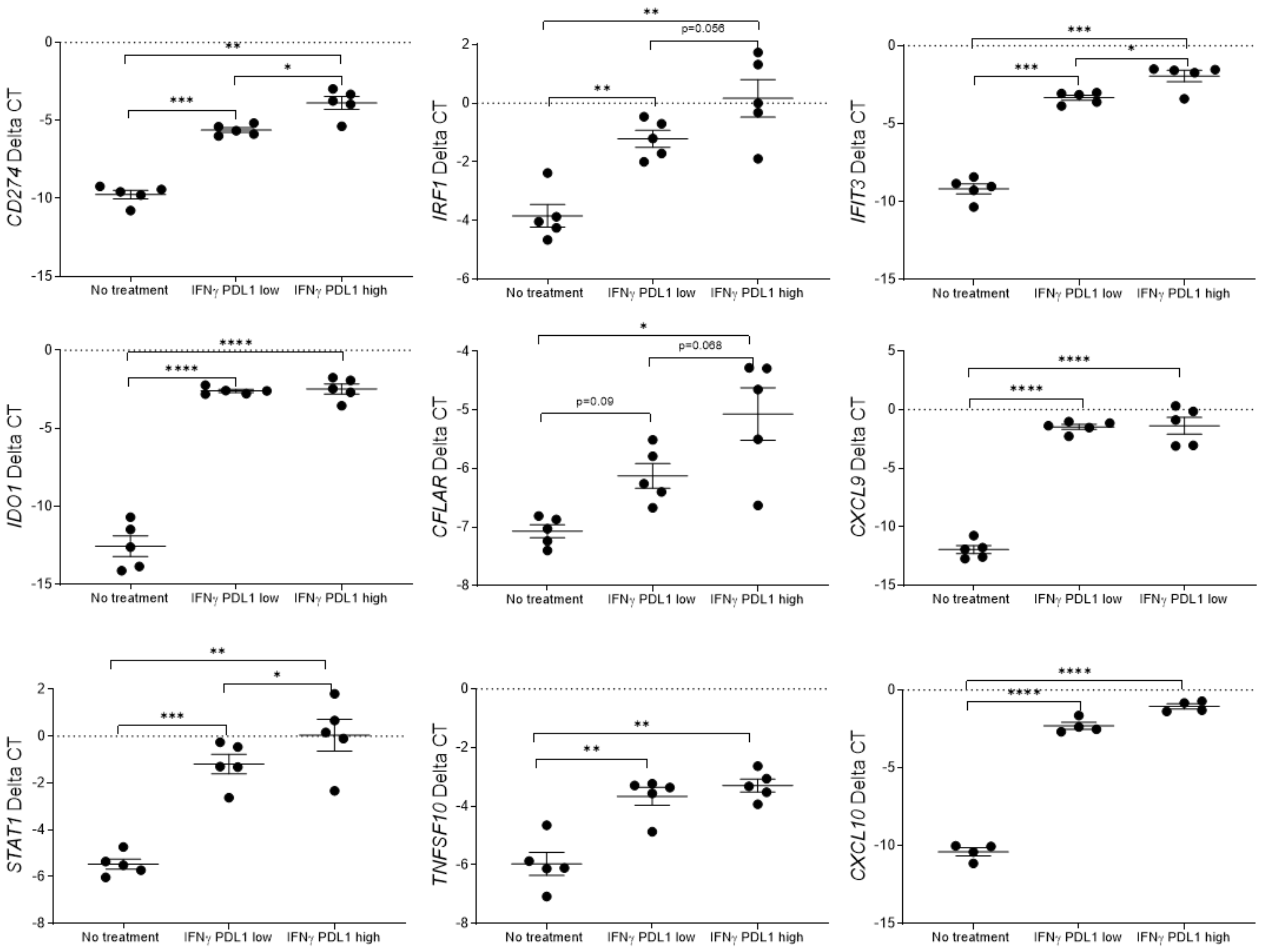

Supplemental Figure 8. Confirmation of transcriptional changes by qPCR in human β cells in response to IFNy. qPCR analysis confirmed induction of select genes identified to be differentially expressed in β cells with IFNy (25 ng/mL) treatment by RNA-seq. Induction of some genes was higher in FACS sorted β cells with higher PD-L1 expression in the presence of IFNγ. N=5 for control and IFNy treatment, except for CXCL10 N=4. DNA was pooled from 3-6 control wells and 5-8 IFNγ treatment wells. Mean +/- SEM. *p≤0.05, ** p≤0.01, *** p≤0.001, **** p≤0.0001 by One-way ANOVA.

Supplemental Table 1: Clinical characteristics of patients with cancers in these studies

	**CPI treated nondiabetic**	**CPI-DM**	**P-values***
Pancreatic enzyme analysis:			
# of patients	N=39	N=22	
Therapy	Combination therapy: N=19<br>Monotherapy: N=20	Combination therapy: N=13<br>Monotherapy: N=9	n.s.
Cancers	27 melanoma, 11 renal cell carcinoma, 1 lung cancer	8 melanoma, 7 lung cancer, 5 renal cell carcinoma, 1 pancreatic cancer, 1 gastrointestinal cancer	
Median age (# male)	60.9 yrs (24)	64 yrs (10)	n.s.
Race	37 White or Caucasian, 2 Other	21 White or Caucasian, 1 Other	n.s.
Ethnicity	37 non-Hispanic, 2 Hispanic or Latino	20 non-Hispanic, 1 Hispanic or Latino, 1 not available	n.s.
Median (range) time (wks) from first CPI treatment	16 (1,173)	15 (2, 83)	n.s
Median (range) time (wks) between lipase check and DM diagnosis	NA	0 (-20, 18)	
CT scan pancreatic volume analysis:			
# of patients (# male)	N=5 (4)	N=13 (7)	
Therapy	Combination therapy: N=2<br>Monotherapy: N=3	Combination therapy: N=10<br>Monotherapy: N=3	n.s.
Cancers	2 melanoma, 3 renal cell carcinoma	7 melanoma, 2 renal cell carcinoma, 3 lung cancer, 1 gastrointestinal cancer	
Median (range) time (wks) from CPI start to posttreatment CT	153 (84, 205)	87 (6, 221)	n.s.
Median (range) time (wks) from DM onset to posttreatment CT	NA	59 (-0.6, 206)	

* By Student’s t-test or Fisher’s exact test as appropriate

Supplemental Table 2: Select genes elevated in immune cells in anti-PD-L1 treated mice by bulk RNA-seq

Gene	P-value	FDR step-up	Fold change
Gzmb	1.2E-5	3.25E-3	3.12
Cxcl9	6.48E-5	0.01	3.60
Cd8a	3.26E-4	0.03	2.04
Pdcd1	4.94E-4	0.04	2.15
Gzma	6.87E-4	0.04	2.82
Fasl	1.19E-3	0.06	2.65
Upregulated but not significant by FDR:			
Cd274	5.23E-3	0.14	2.00
Ifng	8.4E-3	0.18	2.07
Cxcl10	0.01	0.22	3.59
Prf1	0.02	0.25	2.85

Supplemental Table 3: Differentially expressed genes in islet cells from anti-PD-L1 versus anti-CTLA-4 treated mice by bulk RNA-seq

Gene	P-value	FDR step-up	Fold change
Sprr1a	5.27E-11	6.92E-7	81.33
Ankrd13a	5.44E-7	3.57E-3	2.77
Ifit3	4.61E-6	0.02	5.70
Mlkl	8.48E-6	0.02	3.82
Irf1	1.04E-5	0.02	2.17
Slc2a2	1.05E-5	0.02	-2.57
Cd274	1.18E-5	0.02	3.38
Ifit2	1.22E-5	0.02	3.61
Gm37013	1.39E-5	0.02	16.95
Oas1a	1.45E-5	0.02	5.85
Ddit3	2.25E-5	0.02	2.06
Irf7	2.27E-5	0.02	4.44
Ifi44	2.5E-5	0.02	5.77
Gbp2	2.54E-5	0.02	4.98
Gbp7	6.01E-5	0.05	3.42
Cenpj	6.1E-5	0.05	2.88
Fcgbp	6.32E-5	0.05	14.20
Ly6e	6.42E-5	0.05	2.59
Batf2	6.85E-5	0.05	3.52
Isg20	7.08E-5	0.05	2.63
Znfx1	8E-5	0.05	2.10
Fam83d	1.04E-4	0.06	-8.40
Oasl2	1.17E-4	0.06	3.54
Maff	1.23E-4	0.06	3.00
Cxcl10	1.24E-4	0.06	5.45
Usp18	1.26E-4	0.06	2.58
Ifit1	1.3E-4	0.06	3.80
Gbp4	1.31E-4	0.06	4.66
Lin7c	1.34E-4	0.06	-2.55
Rsad2	1.41E-4	0.06	4.30
Mras	1.49E-4	0.06	2.03

Supplemental Table 4: Examples of IFNγ responsive genes upregulated on β cells in main β cell cluster with anti-PD-L1 treatment compared to anti-CTLA-4

Gene	p-value	log2fc
Cxcl10	5.61E-16	2.637051
Cd274	9.81E-15	3.323586
B2m	3.05E-13	0.839975
Stat1	6.64E-12	1.574758
Irf1	6.50E-07	1.748058
Ccl5	8.13E-07	3.614368
Ccl8	1.93E-06	5.744423
Tnfsf10	3.84E-06	2.213323
Slc2a2	2.02E-05	-1.16702
Lgals9	0.000196	2.521372

Supplemental Table 5: Human Islet Donors for Single Cell RNA-seq

Donor	Age	Sex	Race	BMI	Cause of death	HbA1c
1	33	F	Hispanic	32.4	Anoxic secondary to alcohol	4.9%
2	46	M	Hispanic	33	Anoxic event secondary to stroke	5.4%
3	58	M	Caucasian	31.8	Stroke	5.9%

Supplemental Table 6: Human and mouse primers used in qPCR analysis

Gene	Forward	Reverse
Human primers:		
CD274	TGGCATTTGCTGAACGCATTT	TGCAGCCAGGTCTAATTGTTTT
IDO1	GCCAGCTTCGAGAAAGAGTTG	ATCCCAGAACTAGACGTGCAA
STAT1	CGGCTGAATTTCGGCACCT	CAGTAACGATGAGAGGACCCT
IRF1	CTGTGCGAGTGTACCGGATG	ATCCCCACATGACTTCCTCTT
IFIT3	AGAAAAGGTGACCTAGACAAAGC	CCTTGTAGCAGCACCCAATCT
CXCL9	CCAGTAGTGAGAAAGGGTCGC	AGGGCTTGGGGCAAATTGTT
CXCL10	GTGGCATTCAAGGAGTACCTC	TGATGGCCTTCGATTCTGGATT
FAS	AGCTTGGTCTAGAGTGAAAA	GAGGCAGAATCATGAGATAT
TNFSF10	CGTGTACTTTACCAACGAGCTGA	ACGGAGTTGCCACTTGACTTG
CFLAR	GACAGAGCTTCTTCGAGACAC	GCTCGGGCATACAGGCAAAT
ACTB	CATGTACGTTGCTATCCAGGC	CTCCTTAATGTCACGCACGAT
Mouse primers:		
Irf1	ATGCCAATCACTCGAATGCG	TTGTATCGGCCTGTGTGAATG
Slc2a2	TCAGAAGACAAGATCACCGGA	GCTGGTGTGACTGTAAGTGGG
Cxcl10	CCAAGTGCTGCCGTCATTTTC	GGCTCGCAGGGATGATTTCAA
Cxcl9	GGAGTTCGAGGAACCCTAGTG	GGGATTTGTAGTGGATCGTGC
Pdcd1	ACCCTGGTCATTCACTTGGG	CATTTGCTCCCTCTGACACTG
Fasl	TCCGTGAGTTCACCAACCAAA	GGGGGTTCCCTGTTAAATGGG
Ifng	ATGAACGCTACACACTGCATC	CCATCCTTTTGCCAGTTCCTC
Gzmb	CCACTCTCGACCCTACATGG	GGCCCCCAAAGTGACATTTATT
Ido1	CAAAGCAATCCCCACTGTATCC	ACAAAGTCACGCATCCTCTTAAA
Cd274	GCTCCAAAGGACTTGTACGTG	TGATCTGAAGGGCAGCATTTC
Prf1	AGCACAAGTTCGTGCCAGG	GCGTCTCTCATTAGGGAGTTTTT
Actb	GGCTGTATTCCCCTCCATCG	CCAGTTGGTAACAATGCCATGT
